# Comparative diet-gut microbiome analysis in Crohn’s disease and Hidradenitis suppurativa

**DOI:** 10.3389/fmicb.2023.1289374

**Published:** 2023-11-10

**Authors:** Peter Cronin, Siobhan McCarthy, Cian Hurley, Tarini Shankar Ghosh, Jakki C. Cooney, Ann-Marie Tobin, Michelle Murphy, Eibhlís M. O’Connor, Fergus Shanahan, Paul W. O’Toole

**Affiliations:** ^1^Department of Biological Science, University of Limerick, Limerick, Ireland; ^2^APC Microbiome Ireland, University College Cork, Cork, Ireland; ^3^Department of Dermatology, South Infirmary Victoria University Hospital, Cork, Ireland; ^4^School of Microbiology, University College Cork, Cork, Ireland; ^5^Department of Computational Biology, Indraprastha Institute of Information Technology Delhi (IIIT-Delhi), Delhi, India; ^6^Department of Dermatology, Tallaght University Hospital, Dublin, Ireland; ^7^School of Medicine, University College Cork, Cork, Ireland; ^8^Health Research Institute, University of Limerick, Limerick, Ireland

**Keywords:** Hidradenitis suppurativa, Crohn’s disease, gut microbiota, diet, inflammation

## Abstract

**Introduction:**

The chronic inflammatory skin disease Hidradenitis suppurativa (HS) is strongly associated with Crohn’s Disease (CD). HS and CD share clinical similarities and similar inflammatory pathways are upregulated in both conditions. Increased prevalence of inflammatory disease in industrialised nations has been linked to the Western diet. However, gut microbiota composition and diet interaction have not been compared in HS and CD.

**Methods:**

Here we compared the fecal microbiota (16S rRNA gene amplicon sequencing) and habitual diet of previously reported subjects with HS (*n* = 55), patients with CD (*n* = 102) and controls (*n* = 95).

**Results and discussion:**

Patients with HS consumed a Western diet similar to patients with CD. Meanwhile, habitual diet in HS and CD was significantly different to controls. Previously, we detected differences in microbiota composition among patients with HS from that of controls. We now show that 40% of patients with HS had a microbiota configuration similar to that of CD, characterised by the enrichment of pathogenic genera (Enterococcus, Veillonella and Escherichia_Shigella) and the depletion of putatively beneficial genera (Faecalibacterium). The remaining 60% of patients with HS harboured a normal microbiota similar to that of controls. Antibiotics, which are commonly used to treat HS, were identified as a co-varying with differences in microbiota composition. We examined the levels of several inflammatory markers highlighting that growth-arrest specific 6 (Gas6), which has anti-inflammatory potential, were significantly lower in the 40% of patients with HS who had a CD microbiota configuration. Levels of the pro-inflammatory cytokine IL-12, which is a modulator of intestinal inflammation in CD, were negatively correlated with the abundance of health-associated genera in patients with HS. In conclusion, the fecal microbiota may help identify patients with HS who are at greater risk for development of CD.

## 1. Introduction

Hidradenitis Suppurativa (HS) is an incompletely understood, painful chronic autoinflammatory skin disorder of the terminal hair follicle. The prevalence of HS is increasing globally (Vazquez et al., 2013; Ingvarsson, 2017; Alotaibi, 2023) and affects up to 1% of the population (Jemec et al., 1996; Cosmatos et al., 2013; Sung and Kimball, 2013; Jemec and Kimball, 2015; Miller et al., 2016; Alotaibi, 2023). However, estimates have varied widely due to the diversity in methodologies and sources utilised and in the USA it has been suggested that HS is prevalent in 0.1% of the population (Ingram, 2020). This debilitating disease is characterised by the development of abscesses, nodules and fistulae which typically occur at intertriginous sites of the body including the inframammary, axillary, gluteal and inguinal regions (Zouboulis et al., 2015; Saunte and Jemec, 2017; Sabat et al., 2020). Hereditary HS which is characterised by loss of function mutations to genes encoding the subunits of γ-secretase (NCSTN, PSENEN, and PSEN1) accounts for a minority of cases (Moltrasio, 2022) whilst environmental and lifestyle factors are thought to be the main driver. Typically, the first events occurring at the terminal hair follicle include infundibular acanthosis, hyperkeratosis and perifollicular immune cell infiltration (Wolk et al., 2020). It is thought that molecules associated with cell damage and bacteria can initiate inflammation leading to immune cell infiltration that clinically manifests as inflamed nodules and abscesses (Wolk et al., 2020). Epigenetic mechanisms including DNA methylation of miRNA genes has also been linked to development of HS (Radhakrishna, 2022). Importantly, the indigenous microbiota has been implicated in HS pathogenesis. We (McCarthy et al., 2022) and others (Ring et al., 2017) have shown significant alterations in the composition of the skin and faecal microbiota of patients with HS compared to controls. Significantly enriched taxa in the skin microbiota included: *Peptoniphilus lacrimalis*, *Peptoniphilus coxii*, *Anaerococcus murdochii*, *Anaerococcus obesiensis* and *Finegoldia magna* (Ring et al., 2017; McCarthy et al., 2022). *Finegoldia magna* is a pathogenic species that is associated with a pro-inflammatory response—thus likely to contribute to the pathogenesis of HS (Neumann et al., 2020). HS is also associated with co-morbidities including obesity, metabolic syndrome and Crohn’s disease (CD; Sabat et al., 2012; Gold et al., 2014; Chen and Chi, 2019). The concurrent existence of inflammatory/metabolic gut and skin diseases has highlighted the potential involvement of the gut-skin axis as a contributor to HS (Eppinga et al., 2016; Balato et al., 2018; Kam et al., 2020; Lam et al., 2021). CD development is mediated by an altered inflammatory response which is typically characterised by alterations to innate immunity of the intestinal mucosa barrier and remodelling of the extracellular matrix through expression of metalloproteins and adhesion molecules (Petagna et al., 2020). This reshaping of the intestinal microenvironment increases leucocyte migration to areas of intestinal inflammation promoting a TH1 response through production of IL-12 and TNFα cytokines (Petagna et al., 2020). Interestingly, both HS and CD are characterised by shared disease manifestations. The HS faecal microbiome is enriched with *Ruminococcus gnavus* and *Clostridium ramosum* which have been previously detected in the CD microbiota and are associated with the upregulation of the inflammatory cytokine TNFα (Joossens et al., 2011; Hall et al., 2017; Nishino et al., 2018; Lloyd-Price et al., 2019; McCarthy et al., 2022). Other pro-inflammatory pathways are also upregulated in both disease states, mediated by the cytokines IL-6, IL-1, IL-17, IL-12 and IL-23 (Duerr et al., 2006; Schlapbach et al., 2011). A dysfunctional Th1 inflammatory response is a key characteristic of both HS and CD (Vossen et al., 2018; Grand et al., 2020). Furthermore, both HS and CD are characterised by shared manifestations such as sinus tract and abscess formation (Van Der Zee et al., 2014). Given the links and overlapping features of these inflammatory diseases it is unsurprising that patients with HS have a significantly higher risk for the development of CD (Deckers et al., 2017; Egeberg et al., 2017). Like HS (McCarthy et al., 2022), alterations to the gut microbiota in patients with CD has been well documented (Halfvarson et al., 2017; Pascal et al., 2017; Franzosa et al., 2018; Yilmaz et al., 2019). Although some of these changes in microbiota composition are likely secondary to intestinal inflammation, specific pro-inflammatory taxa have been implicated in CD through a number of clinical and experimental studies (Bernstein and Forbes, 2017; Nishida et al., 2017; Sheehan and Shanahan, 2017; Zuo and Ng, 2018; Ryan et al., 2020). It is currently unknown whether inflammation driving the development of HS occurs first at the epidermis or is extracutaneous. If extracutaneous inflammation occurs first, the intestinal microbiota could play an important role in its pathogenesis. Furthermore, given that a significant number of HS patients eventually present with CD, the microbiota may also have a role in the development of CD in patients with HS. To date, we are unaware of any study that has compared the gut microbiota of patients with HS with that of patients with CD.

The global increase in HS prevalence is most pronounced in industrialised countries, consistent with trends for other metabolic and inflammatory diseases including CD (Roda et al., 2020). This increased prevalence is also linked to consumption of the Western diet, which is low in dietary fibre and high in sugars, saturated fat and dairy (Cordain et al., 2002; Simopoulos and DiNicolantonio, 2016; Khan and Chang, 2022). Diet is now being recognised as an effective approach to help mitigate the burden of inflammatory disease. It has been suggested that dairy products (William Danby, 2015; Silfvast-Kaiser et al., 2019; Khan and Chang, 2022) and brewer’s yeast (Silfvast-Kaiser et al., 2019; Khan and Chang, 2022) can aggravate HS symptoms. It is important to note that the reported associations between diet and HS are controversial. The few studies examining dairy and/or brewers’ yeast in HS used small sample sizes and did not use formally validated assessment methods (placebo control; Cannistrà et al., 2013; Colboc et al., 2016). Currently it is not clear what role diet plays in HS, and little is known about day-to-day dietary intake patterns in patients undergoing treatment.

To address these questions, we undertook a study to compare faecal microbiota composition and habitual diet in patients with HS to patients with CD and healthy controls.

## 2. Materials and methods

### 2.1. Overview of study population

The study population for this analysis included 55 individuals with HS who were recruited as previously reported (Table 1; McCarthy et al., 2022). For comparative analysis we included data for 102 patients with CD obtained from a previously published dataset (Table 1; Clooney et al., 2021). The control group included 95 individuals with no known medical condition, the samples for which were obtained from both studies (Table 1; Clooney et al., 2021; McCarthy et al., 2022). Patient data obtained from Clooney et al. (2021) included multiple longitudinal samples for some individuals. Whilst different datasets were used for analysis in this study, sample collection procedures, DNA extraction and 16S PCR protocols as well as sequencing methodology (Illumina MiSeq 2 × 300 bp chemistry) were uniform across both studies. Dietary data was previously collected using a food frequency questionnaire (FFQ) which we obtained on request from the authors.

**Table 1:** Study population and sample sizes.

	Patients (*n*)	Samples	Dataset
Controls	95	164	Clooney et al. (2021)^*^ and McCarthy et al. (2022)^*^
Crohn’s disease (CD)	102	212	Clooney et al. (2021)
Hidradenitis suppurativa (HS)	55	55	McCarthy et al. (2022)

^*^Controls had *n* = 30 patients (30 samples) from McCarthy et al. (2022) whilst rest of the controls was *n* = 72 (182 samples) obtained from Clooney et al. (2021).

### 2.2. Pre-processing of 16S amplicon sequence reads

16S rRNA amplicon raw reads sequenced from faecal samples were obtained from two previously published datasets which are publicly available on the European Nucleotide Archive (ENA) under accession number PRJEB43835 (McCarthy et al., 2022) and PRJNA414072 (Clooney et al., 2021; Table 1). Firstly, raw reads were processed using the filterAndTrim function in the DADA2 package with the parameters trimLeft = 19, maxEE = 2, truncLen = 240 (version 1.18; Callahan et al., 2016). We used the forward reads only for analysis owing to the lower quality present in the reverse reads, which is known to negatively impact sample inference downstream in the DADA2 pipeline. Read dereplication, learning of the error rates, and sample sequence variant inference with pooled samples followed by the construction of amplicon sequence variant (ASV) table and removal of chimaeras were performed using DADA2. Taxonomic assignment of reads was carried out using the SILVA database (v138.1) also in DADA2 (Quast et al., 2013). In order to remove any dataset specific noise (study effect) we conducted all analysis at the genus level. In addition, we also applied a quality filtering step removing rare genera from the analysis, keeping those present in 10% or more of samples.

### 2.3. Biostatistical analysis

All biostatistical analysis was carried out in Rstudio (version 4.1.1; RStudio Team, 2020). Both alpha (α) diversity and beta (ß) diversity (Bray–Curtis dissimilarity) were calculated using the phyloseq (version 1.36; McMurdie and Holmes, 2013) and the vegan (version 2.7; Oksanen et al., 2022) packages in R. To test for differences in ß-diversity between the groups we used Permutational Analysis of Variance (PERMANOVA) whilst controlling for the study effect and patient identifier. Principal component analysis (PCoA) was used to visualise differences in microbiota composition and conducted using the dudi.pco and s.class functions of the ade4 (version 1.7) package (Thioulouse et al., 2018). To perform quantitative comparative evaluation of differences observed in microbiota composition we use a median centroid testing methodology as previously employed by us (Cronin et al., 2022). This metric is obtained whereby the median PCoA coordinates of a specific group are calculated, and the distance of all other samples from this point is subsequently determined. In order to establish significantly differentially abundant genera between groups we used Analysis of Compositions of Microbiomes with Bias Correction (ANCOMBC; version 2.2.0; Breiman, 2001). The machine learning random forest approach was used to identify the most discriminatory features when comparing different groups using the randomForest package in R (version 4.7; Breiman, 2001). The approach used to identify the Co-Abundance Groups (CAGs) was carried out as previously reported by us (Flemer et al., 2017; Cronin et al., 2022). Briefly, we used ward-D2 clustering on the genera-level spearman correlation matrix and CAGs were subsequently identified by cutting the generated dendrogram to obtain four distinct clusters of genera. Differences in overall habitual diet were visualised on a PCoA using a Kendall tau distances. Spearman correlations were carried out using the corr.test function of the psych package (version 2.3.3; Revelle, 2017). Logistic regression was used to identify dietary ingredients, drugs or clinically relevant metadata factors which co-varied between groups. Associations between microbial genera and inflammatory markers were tested using the CCREPE package (version 1.36). Heatmaps were generated using the heatmap.2 function in the gplots package (version 3.3.1). Network plots were generated using the gephi (version 0.10.1; Bastian et al., 2009). Chord diagrams were generated using the chordDiagram function in the circlize package (Gu et al., 2014). All other graphics were produced using the ggplot2 package (version 3.4.2). Furthermore, where rank normalised genera abundances were used they were calculated using the formula [rank(x)-(min(rank(x)))/max(rank(x))-min(rank(x))]. Statistical significances (unless otherwise stated) were calculated by employing the non-parametric Kruskal–Wallis’ test with Dunn’s *post hoc* test where three or more groups were being compared. Where only two groups were compared, a Wilcoxon’s test was used. All *p-*values presented in this study were FDR corrected using the Benjamini Hochberg method.

### 2.4. Inflammatory marker measurement

Serum samples corresponding to patients with HS from McCarthy et al. (2022) dataset were analysed for several markers of inflammation. For measurement of adipokines (leptin and adiponectin), growth-arrest specific 6 (Gas6), TNFα, IL-6, and C-reactive protein (CRP), venous blood was drawn into 2.5 mL vacuette tube (red top) which contained a serum separator clot activator (Grenier Bio-One International). Serum was allowed to clot at room temperature for approximately 30 min. Subsequently the serum was separated by centrifugation (5,000 rpm for 15 min). Serum concentrations for each inflammatory marker was determined using an enzyme-linked immunosorbent assay (ELISA; Protein Simple-Simple Plex Cartridge Kit, BioTechne). Samples were prepared and loaded into the cartridge according to a standard procedure provided by the manufacturers with all steps in the immunoassay procedure automated by the Ella instrument (Biotechne). All ranges for detection and quantification are provided in detail for each of the proteins evaluated from the company website documentation. Human complement C5a levels in serum were also measured using an ELISA method (Abcam—ab193695). ELISA based calprotectin assay (R&D Inc. S100A8/S100A9) was performed with faecal material from each patient with HS as per the manufacturers protocol.

## 3. Results

### 3.1. Microbiota composition in most patients with HS differs significantly from that of patients with CD

**Figure 1:**
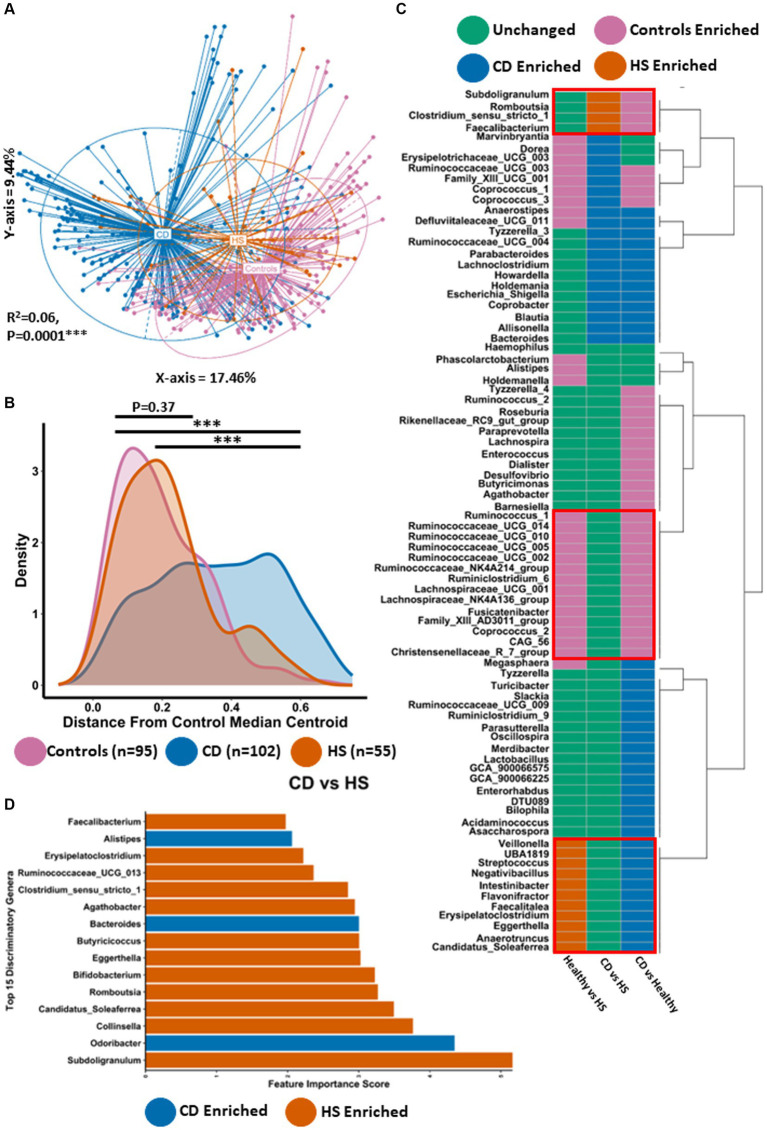
Microbiota composition in HS is significantly different to CD for most individuals. **(A)** Principal Component Analysis (PCoA) of β-diversity (Bray–Curtis dissimilarity) at the genus level (16S rRNA gene amplicon profiles). The *P*-value (0.0001) obtained using a PERMONOVA shows there is statistically significant microbiome separation between the groups even after controlling for the study effect and patient identifier as confounders. The eigen values are also reported which show the variation reported in the X-axis (17.46%) and Y-axis (9.44%) of the PCoA. **(B)** Using the PCoA coordinates from **(A)** the median control centroid was calculated and the distance of all samples from those coordinates was subsequently determined and displayed here as a density plot. Density is displayed on the Y-axis whilst the actual reported distance from the control median centroid is displayed on the X-axis. Kruskal-Wallis with Dunn’s *post hoc* test was used to determine significant differences between the groups for this distance measure. The annotations used for *p*-values are *p* < 0.05 *; *p* < 0.01 **; *p* < 0.001***. All displayed *p*-values are FDR corrected. **(C)** ANCOMBC differential abundance analysis was sued to determine significantly differentially abundant genera between the groups controlling for the study effect and patient identifier. Each colour signifies whether that specific genera was unchanged (green), higher in controls (purple), higher in patients with CD (blue) or higher in patients with HS (orange). **(D)** Barplot depicting the top 15 most discriminatory genera from a machine learning random forest classifier comparing HS and CD. The colour indicates whether that genera is found in higher abundance in HS or CD.

Principal coordinate analysis (PCoA) was carried out using the Bray–Curtis dissimilarity β-diversity measure to investigate differences in microbiota composition at the genus level (Figure 1A). Given that the study population contained data from different sequencing runs as well as multiple longitudinal samples from some individuals, we adjusted for the study effect and patient identifier as confounders. Using this method, we identified statistically significant microbiome separation between all groups (PERMANOVA FDR-corrected *p* < 0.0001: R^2^ = 0.06). Interestingly, large differences in microbiota composition were observed between patients with HS and patients with CD (Pairwise PERMANOVA FDR-corrected *p* < 0.001: R^2^ = 0.08). In line with what was previously reported (Clooney et al., 2021; McCarthy et al., 2022), patients with either HS (Pairwise PERMANOVA FDR-corrected *p* < 0.001: R^2^ = 0.07) or CD (Pairwise PERMANOVA FDR-corrected *p* < 0.001: R^2^ = 0.07) both had a significantly different genus-level microbiota composition to controls. Statistically significant microbiome separation (Bray–Curtis dissimilarity) was also observed at the ASV level (PERMANOVA FDR-corrected *p* < 0.0001: R^2^ = 0.04) after controlling for the study effect and patient identifier as confounders (Supplementary Figure 1). Next, we conducted a quantitative comparative evaluation of the reported differences in microbiota composition using a median centroid testing methodology calculated from the PCoA of Bray–Curtis dissimilarity in Figure 1A. This calculates the similarity between the microbiota of all faecal samples and the median PCo1 (X-axis) and PCo2 (Y-axis) coordinates (presented in Figure 1A) of the control group. Ultimately this calculation provides the distance between each sample and the control group median centroid. Statistically significant differences were observed between the HS and CD patient groups for this measure (Figure 1B). The majority of patients with HS harboured a microbiota that was a much smaller distance from the control median centroid than those with CD. In keeping with this observation, no significant differences could be found when comparing the HS and control study groups (*p* = 0.37). Interestingly, there was no significant difference in two α-diversity measures (Shannon and Simpson) between HS and CD (Supplementary Figures 1A,B). However, patients with HS or CD had a significantly lower microbiota α-diversity when compared to controls (Supplementary Figures 1A,B).

Given the differences in microbiota composition observed between HS and CD, we wanted to determine what specific genera were significantly differentially abundant between the groups. Thus, we conducted ANCOMBC differential abundance analysis controlling for the study effect (Figure 1C; Supplementary File 1). In total, 24 genera were significantly enriched or depleted between patients with HS and patients with CD. Of these genera, 4 were significantly enriched in the HS microbiota (Figure 1C). *Faecalibacterium* was the most significantly elevated of these taxa followed by *Subdoligranulum*, *Romboustia* and *Clostridium_senu_stricto_1, respectively* (Supplementary Figure 2). 20 genera were significantly more abundant in patients with CD compared to those with HS (Figure 1C; Supplementary Figure 2). Of these 20 taxa, *Defluviitaleaceae_UCG_011* and *Marvinbryantia* were the most significantly enriched in the CD microbiota. Other genera in this list included *Bacteroides* and *Escherichia_Shigella*, both of which have been associated with CD in previous studies. Whilst clear differences exist in the faecal microbiota of both HS and CD, we find here that the microbiome of both inflammatory disease states can be characterised by a shared set of 11 genera which are significantly enriched compared to the control group (Figure 1C; Supplementary Figures 3, 4). This core set of inflammatory disease associated genera included *Streptococcus*, *Veillonella*, *Eggerthella* and *Anaerotruncus*, amongst others. The microbiota of HS and CD was also characterised by a shared set of depleted taxa compared to controls (Figure 1C; Supplementary Figures 3, 4). This set of taxa included multiple genera in the *Ruminococcaceae* and *Lachnospiraceae* families as well as genera such as *Coprococcus_2* and *Christensenellaceae_R_7_group*.

A powerful approach for analysing and predicting complex interactions within microbiome data, particularly in the context of comparing disease states to healthy conditions, is the implementation of the machine learning algorithm Random Forest. Using this method to compare the faecal microbiota of HS and CD we were able to construct a predictive model with an error rate of 9% (91% accuracy). Interestingly, we found that the HS enriched taxon *Subdoligranulum* was the most important discriminatory taxon in classifying between the two inflammatory diseases (Figure 1D). Of the top 15 most discriminatory genera in this random forest classifier model, 12 genera were higher in abundance in patients with HS whilst only 3 were found in a higher abundance in those with CD (Figure 1D). For patients with HS this random forest classifier predicted 41% of patients as having a microbiota composition that resembles CD. Comparing the HS faecal microbiota to controls using this same methodology predicted that 43% of HS patients were classified as having a normal microbiota composition. Overall, this second model also exhibited high accuracy as the reported error rate was 14% (86% accurate). The HS enriched taxa *Eggerthella* and *Erysipelatoclostridium* were the most discriminatory genera in classifying between HS and healthy study populations (Supplementary Figure 5; Supplementary File 1). Other discriminatory genera (higher in controls) at the top of this list included members of the *Ruminococcaceae* family as well as *Coprococcus_1*, *Alistipes* and *Butyricimonas*. For context, repeating the random forest machine learning method but this time comparing the healthy and CD study population also resulted in a model with a low error rate of 12% (88% accuracy; see Supplementary Figure 6 for the top 15 most discriminatory genera).

Thus, whilst HS and CD microbiota share a set of common enriched or depleted genera compared with the control microbiota, there are significant differences in microbiota composition between the groups. However, the machine learning random forest approach indicates that some, but not all patients with HS have a similar microbiota composition to CD.

### 3.2. HS and CD have different distinctive co-abundance group microbiota profiles

**Figure 2:**
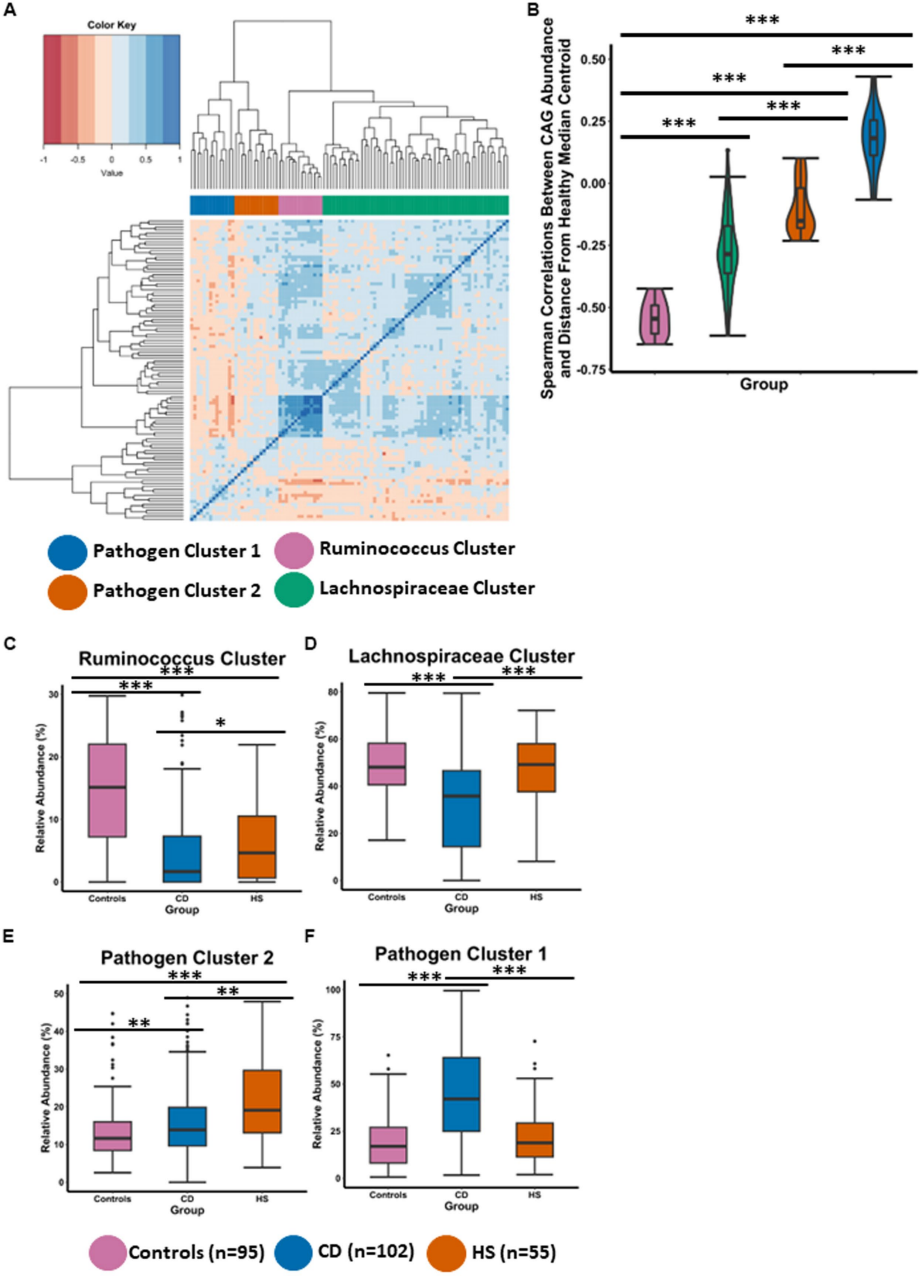
HS and CD are characterised by different distinctive co-abundance group (CAG) microbiota profiles. **(A)** Heatmap illustrates the ward.d2 clustering of Spearman correlation coefficients representing the relative abundance of genera in the faecal microbiota of individuals included in this study. Each distinct co-abundance group (CAG) is visually distinguished by its corresponding colour as indicated by the legend to the right. **(B)** Violin plot showing the spearman correlations between CAG abundance and the control median centroid which was previously determined for Figure 1B. **(C–F)** Boxplots showing the relative abundance (%) of each CAG across the three groups. Statistical significance was calculated using Kruskal-Wallis with Dunn’s *post hoc* test. The annotations used for *p*-values are *p* < 0.05 *; *p* < 0.01 **; *p* < 0.001***. All displayed *p*-values are FDR corrected.

To further understand compositional microbiota differences between the HS and CD inflammatory diseases, we employed a co-abundance group (CAG) analysis, which is an approach previously used successfully in our group (Flemer et al., 2017; Cronin et al., 2022). CAGs are clusters of microbial taxa that consistently occur together and exhibit similar abundance patterns across multiple samples. Briefly, the CAGs or clusters are determined using Spearman correlations of genera abundances followed by hierarchal clustering. CAGs provide further insight into the co-occurrence and interactions amongst different microbial genera within a community. Using this method, we identified four CAGs which we named based on their composition (Figure 2A), specifically, *Ruminococcus cluster*, *Lachnospiraceae cluster*, *Pathogen cluster 1* and *Pathogen cluster 2*. The detailed taxon memberships of each CAG are shown in Supplementary File 2.

To statistically test if a CAG was associated with a healthy microbiota composition, we measured Spearman correlations between CAG relative abundance and distance from the control median centroid position which was previously calculated for Figure 1B. Members of the *Ruminococcus cluster* were observed to have strong negative correlations with the distance from the control median centroid (Figure 2B). This indicates that the *Ruminococcus cluster* is associated with a microbiota composition characteristic of controls in this study, as the genera in this cluster have a higher abundance as a function of shorter distance from the control median centroid. The *Lachnospiraceae cluster* was significantly different to the *Ruminococcus cluster* for this measure (Figure 2B). However, the abundance of members in the *Lachnospiraceae cluster* were recorded as negatively correlating with the distance from the control median centroid indicating that this CAG is also health associated. It is also important to note that both CAGs associated with a normal (control) microbiome comprise genera which are thought to be putatively beneficial and capable of fibre-fermentation. Both CAGs (*Ruminococcus cluster* and the *Lachnospiraceae cluster*), were significantly different to *Pathogen cluster 1* and *Pathogen cluster 2, respectively* (Figure 2B). Members of the *Pathogen cluster 1* were observed to have strong positive correlations with the distance from the control median centroid. Similar findings were observed for *Pathogen cluster 2* albeit to a lesser degree. These findings indicate that *Pathogen cluster 1* and *Pathogen cluster 2* are not associated with a normal microbiota composition as the abundance of their members is found at a lower level the larger the distance from the control median centroid.

The findings presented in Figure 2B were strengthened when we compared the abundance of the CAGs across the three study groups (Figures 2C–F). The microbiota composition in controls was characterised by a high abundance of the *Ruminococcus cluster* and the *Lachnospiraceae cluster* as well as a low abundance of *Pathogen cluster 1* and *Pathogen cluster 2* (Figures 2C–F). Interestingly, for the *Ruminococcus cluster*, patients with HS had a significantly higher level than patients with CD (Figure 2C). Both inflammatory disease groups were observed to have a significantly lower abundance of this CAG when compared to individuals in the control group. Patients with HS also had a significantly higher level of the health associated *Lachnospiraceae cluster* when compared to patients with CD (Figure 2D). Interestingly, no significant difference was detected between the HS and control study groups for the abundance of the *Lachnospiraceae cluster* or *Pathogen cluster 1* (Figures 2D,F). CD had the highest abundance of *Pathogen cluster 1* and this was found to be significantly different to patients with HS as well as controls. Patients with HS had the higher abundance of *Pathogen cluster 2* which was significantly higher than patients with CD and controls (Figure 2E). Patients with CD also maintained a significantly higher level of this cluster compared with the control group.

Thus, CAG analysis reveals different microbiome profiles between the groups. Although patients with HS have a higher level of the health associated *Ruminococcus cluster* and the *Lachnospiraceae cluster* than patients with CD, their faecal microbiota is characterised by a high abundance of *Pathogen cluster 2*. Members of this CAG include the putatively pathogenic *Bilophila*, *Dialister*, *Parabacteroides* and *Anaerostipes*, amongst others (Supplementary File 2). The CD faecal microbiota is characterised by a high abundance of Pathogen cluster 1 whose members include a different set of pathogenic genera such as *Streptococcus*, *Escherichia_Shigella*, *Enterococcus* and *Veillonella* (Supplementary File 2).

### 3.3. Similar habitual Western diet in patients with HS and those with CD

**Figure 3:**
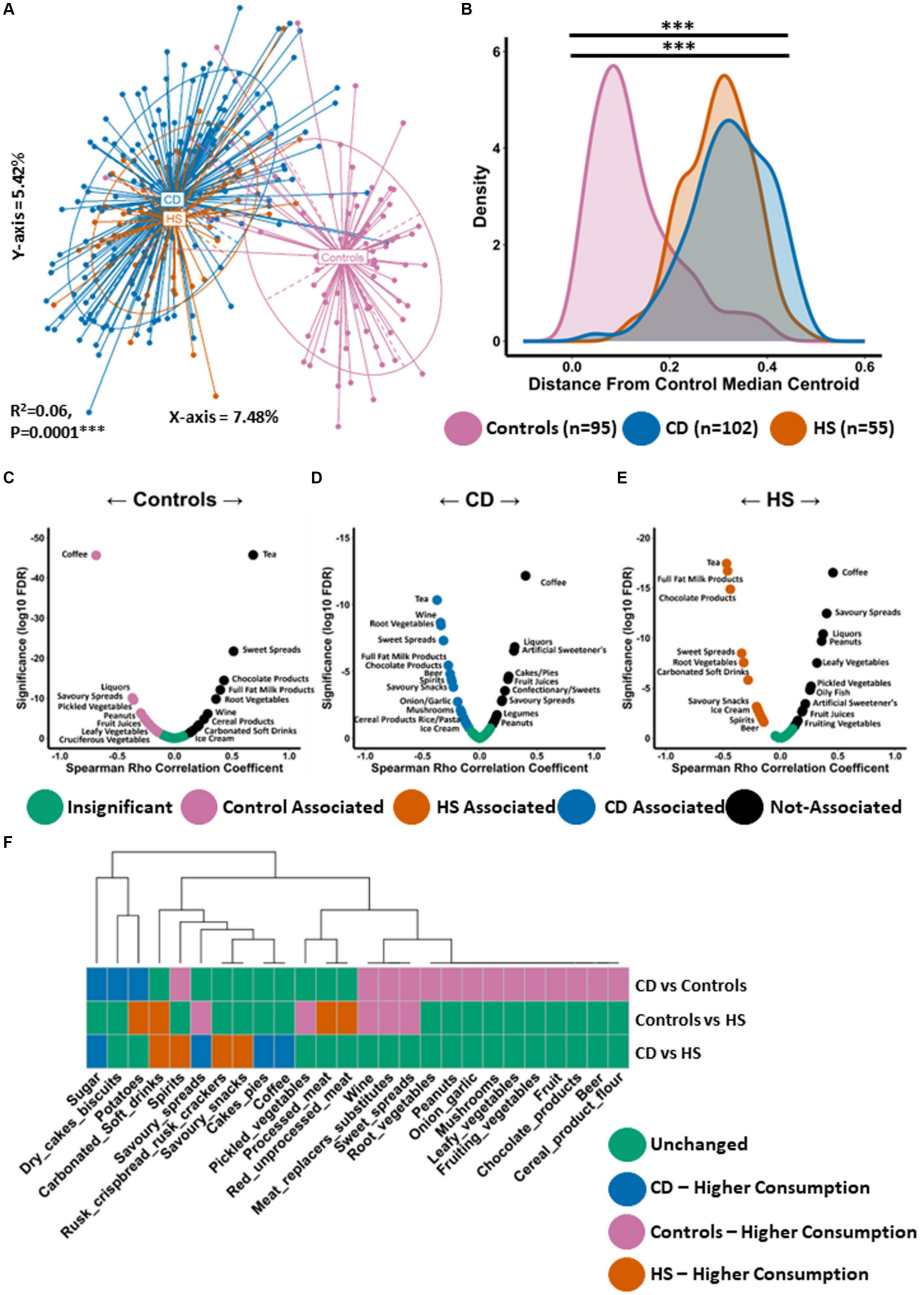
Habitual diet of patients with HS resembles the Western diet similar to patients with CD. **(A)** Principal Component Analysis (PCoA) of habitual dietary profiles (Kendall tau distance) based on daily frequency of consumption. The P Value (0.0001) obtained using a PERMONOVA shows there is statistically significant separation between the groups. The eigen values are also reported which show the variation reported in the X-axis (7.48%) and Y-axis (5.24%) of the PCoA. **(B)** Using the PCoA coordinates from **(A)** the median control centroid was calculated and the distance of all samples from those coordinates was subsequently determined and displayed here as a density plot. Density is displayed on the Y-axis whilst the actual reported distance from the control median centroid is displayed on the X-axis. Kruskal-Wallis with Dunn’s *post hoc* test was used to determine significant differences between the groups for this distance measure. The annotations used for *p*-values are *p* < 0.05 *; *p* < 0.01 **; *p* < 0.001***. All displayed *p*-values are FDR corrected. **(C,D)** Volcano plot showing spearman correlations between the consumption of dietary ingredients and **(C)** the median control centroid, **(D)** the CD median centroid and **(E)** the HS median centroid. A significant negative correlation indicates that a specific dietary ingredient is associated with the median pattern of that patient group as its consumption increase the shorter the distance to the median centroid. Significant positive correlations indicate that a specific food item is not associated with the median dietary of that patient group as it consumption increases the larger the distance from the median centroid. **(F)** Wilcoxon test was conducted to determine what food items were differentially consumed between each pairwise comparison and shown in a colour coded heatmap. Each colour signifies whether that specific genera was unchanged (green), higher in controls (purple), higher in patients with CD (blue) or higher in patients with HS (orange).

We conducted PCoA using the Kendall tau distance measure to investigate differences in habitual diet (daily frequency of consumption) of the study datasets (Figure 3A). We observed statistically significant separation between the groups (PERMANOVA FDR-corrected *p* < 0.0001: R^2^ = 0.06). Habitual diet in patients with HS was significantly different to controls. This finding was confirmed when we measured the similarity (distance) between individuals daily dietary pattern and the coordinates of the control median centroid as calculated from Figure 3A. As expected, patients with HS were a significantly larger distance from the median centroid than controls (Figure 3B). Interestingly, the HS habitual diet was not significantly different to that of CD (Figures 3A,B).

Given the large difference in habitual diet between patients with HS and controls, we wanted to establish what specific dietary ingredients were most or least associated with the median dietary pattern of each group. We conducted Spearman correlation analysis between the consumption levels of each dietary ingredient with the distance from the median centroid of each study group respectively, as calculated from Figures 3A,C–E and Supplementary File 3. As expected, the control group was observed to have a strong negative correlation with the distance from the control median centroid for a number of food items considered to be health promoting and high in dietary fibre (Figure 3C). This list included a variety of vegetables (cruciferous, leafy and pickled) as well as fish (Figure 3C; Supplementary File 3). Dietary ingredients which were least associated (positive correlation) with controls using this approach included chocolate products, ice-cream and carbonated soft drinks, all of which are elements of the Western diet. In total, 29 different food types significantly correlated with the distance from the control median centroid (Supplementary File 3). Using this approach, an opposing trend was identified for CD (Figure 3D; Supplementary File 3) and HS (Figure 3E; Supplementary File 3). When exploring habitual dietary patterns in HS, we found 36 individual food items to significantly correlate with the distance from the HS median centroid (Supplementary File 3). A number of food items whose consumption is typical for the Western diet were found to negatively correlate with the distance from the HS median centroid (Figure 3E), including processed meat, sugar, carbonated soft drinks, ice-cream and chocolate. Several alcoholic beverages were also found to be associated with the HS diet including beer, wine and spirits. Dietary ingredients which were least associated with the HS diet (positive correlation) included fish, a variety of vegetables (pickled, leafy and fruiting) as well as fruit (Figure 3E; Supplementary File 3).

Next, we identified the specific food items that were differentially consumed between the groups. We performed a Wilcoxon test for all individual pairwise comparisons, the full results of which can be found in Supplementary File 3. Although overall habitual diet was similar between HS and CD, with both conditions being associated with a frequency of consumption pattern typical of the Western diet, there were a small number of food items differentially consumed. For example, patients with CD consumed significantly more sugar and cakes/pies than patients with HS (Figure 3F). The HS study group was found to consume larger quantities of carbonated soft drinks and alcohol (spirits), for example (Figure 3F). Compared to controls, patients with HS were found to consume significantly more carbonated soft drinks and processed meat (Figure 3F). Furthermore, patients with HS consumed significantly less pickled vegetables and meat replacer substitutes. The latter is likely an indication of vegetarianism amongst some individuals within the control study population.

Thus, the dietary pattern in patients with HS is a Western type diet, similar to that linked with CD. Dietary ingredients found to positively associate with HS were high in sugar and saturated fat. In addition, food items least associated with HS included vegetables and fruit which are high in dietary fibre and health promoting.

### 3.4. Some patients with HS have a microbiota configuration similar to that of patients with CD

In a previous study, we calculated the relatedness between samples (HS and controls) based on their microbiome composition (ASV level) using Spearman’s rank correlation coefficient and subsequent hierarchical clustering with the Ward2 method (McCarthy et al., 2022). This hierarchical clustering revealed the presence of two HS microbiome clusters. The first cluster was composed exclusively of patients with HS. The second cluster was comprised of healthy controls as well as the remaining patients with HS. When we compared these two distinct microbiome groups of patients with HS, we found that one group was significantly older and had a lower microbiome α-diversity (McCarthy et al., 2022). At the time of publication, no other factor was detected as co-varying with the observed differences in microbiota composition. Building on this finding, we showed above using the machine learning (random forest) approach that 41% of patients with HS were predicted as having a microbiota composition resembling CD (Figure 1E). Furthermore, we also established using this same approach that 42% of patients with HS were predicted as having a microbiota composition similar to controls (Supplementary Figure 5). With respect to the findings from both McCarthy et al. (2022) and this current study, we wanted to further investigate differences in microbiota composition in patients with HS.

**Figure 4:**
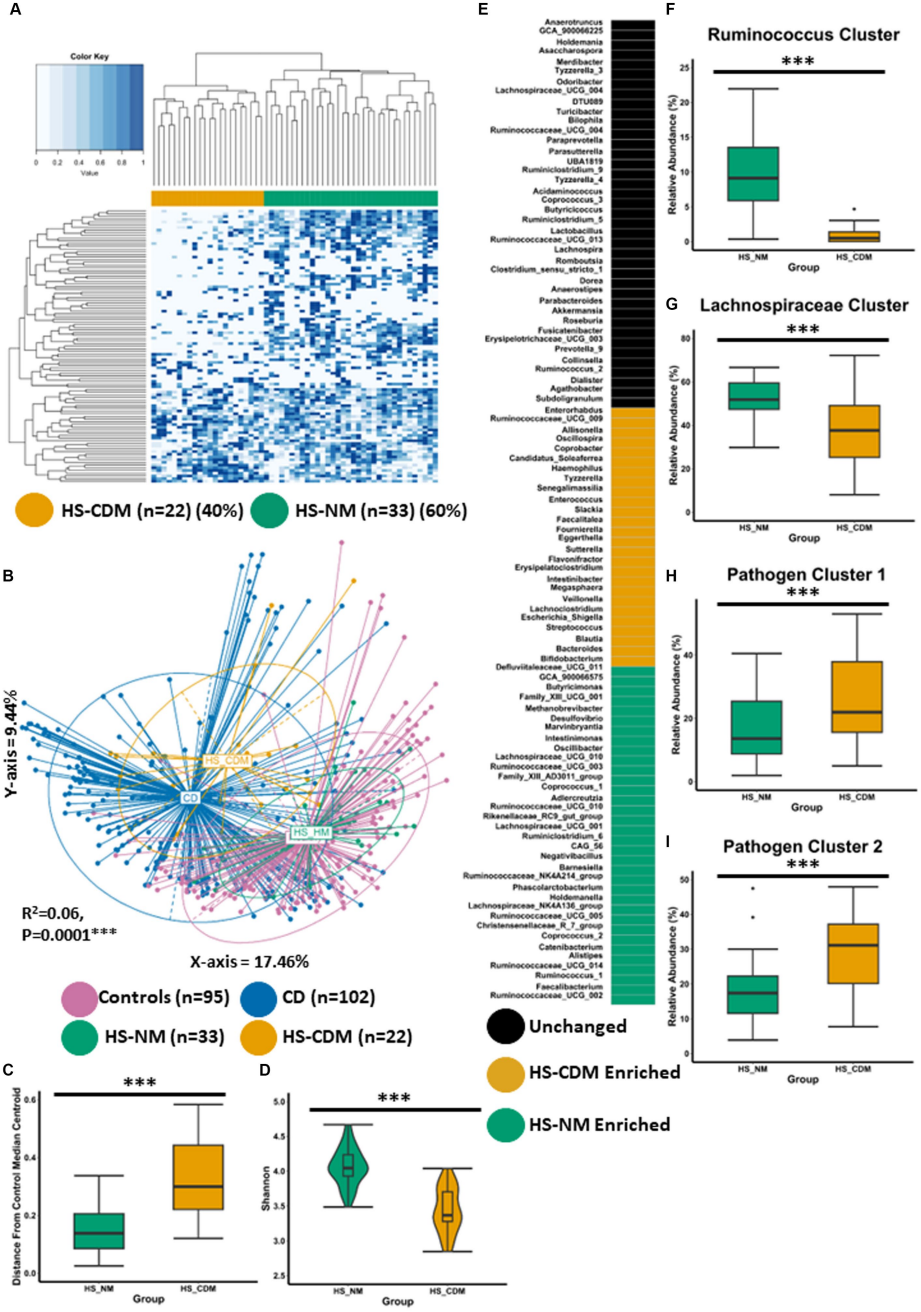
Some patients with HS have a microbiota configuration similar to patients with CD. **(A)** Heatmap showing the genus profiles of all patients with HS. Through Ward.d2 clustering two genus level microbiome groups were identified labelled HS-NM (HS-Healthy-like microbiota; green) and HS-CDM (HS-Crohns disease-like microbiota; yellow). **(B)** Principal Component Analysis (PCoA) of β-diversity (Bray–Curtis dissimilarity) at the genus level (16S rRNA gene amplicon profiles). The *p*-value (0.0001) obtained using a PERMONOVA shows there is statistically significant microbiome separation between the groups even after controlling for the study effect and patient identifier as confounders. The eigen values are also reported which show the variation reported in the X-axis (17.46%) and Y-axis (9.44%) of the PCoA. **(C)** Using the PCoA coordinates from **(A)** the median control centroid was calculated and the distance of samples (HS-HM and HS-CDM) from those coordinates was subsequently determined and displayed here as a box plot. **(D)** Boxplot showing Shannon microbiome α-diversity between the groups. **(E)** ANCOMBC differential abundance analysis was sued to determine significantly differentially abundant genera between the groups. Each colour signifies whether that specific genera was unchanged (black), higher in HS-HM (green), higher HS-CDM (yellow). **(F–I)** Boxplots showing the relative abundance (%) of each CAG across the two groups of patients with HS. Wilcoxon test was used to determine significant differences between the groups. The annotations used for *p*-values are *p* < 0.05*; *p* < 0.01**; *p* < 0.001***. All displayed *p*-values are FDR corrected.

We used an improved approach for identifying differences in microbiota composition within a single study group which was in keeping with findings (in this current study) detected at the genus level. We calculated the relatedness between samples solely from patients with HS (not including healthy controls) using ranked genera abundances and subsequent Ward2 clustering (Figure 4A). Using this approach, we identified two groups which separated based on genus-level faecal microbiota composition. The first group represented the majority of patients with HS (60%) whilst the second group represented 40% of the study group (Figure 4A). Next, we wanted to better understand how these groups compared to the microbiota of controls and patients with CD. We conducted PCoA of β-diversity (Bray–Curtis dissimilarity) at the genus level showing statistically significant microbiome separation between the groups (PERMANOVA FDR-corrected *p* < 0.0001: R^2^ = 0.06). We observed that one cluster of patients with HS had a microbiota composition most similar to the normal microbiota composition detected in controls and was subsequently named HS-NM (HS-Normal-like microbiota; *n* = 33; Figure 4B). The second group of patients with HS compromising 40% of the population was observed to have a microbiota composition most similar to patients with CD and was subsequently named HS-CDM (HS-Crohn’s disease-like microbiota; *n* = 22; Figure 4B). This finding was further corroborated with the observation that the HS-CDM patient cluster was a significantly greater distance from the control median centroid than the HS-NM group (Figure 4C). Patients in the HS-NM group were also shown to have a significantly higher microbiome α-diversity (Figure 4D; Supplementary Figures 7A–C).

To identify the specific genera that are significantly differentially abundant between HS microbiome patient clusters, we conducted ANCOMBC differential abundance analysis whereby we identified 60 genera as being significantly different between the two groups (Figure 4E; Supplementary File 4). 34 genera were identified as being significantly higher in the HS-NM patient group as compared to HS-CDM. Many of these genera had also been identified as being significantly higher in controls when compared to patients with CD (Figure 1C). This included several members of the *Ruminococcaceae* and *Lachnospiraceae* families as well as, *Faecalibacterium*, *Buyricimonas* and *Coprococcus_1* (Figure 4E). Meanwhile, patients in the HS-CDM group were found to have significantly higher levels of several genera typically enriched in CD, including *Escherichia_Shigella*, *Bacteroides*, *Enterococcus*, *Streptococcus* and *Veillonella* (Figure 4E). When we compared CAG abundance between HS-NM and HS-CDM the results were strikingly similar to what we observed when comparing controls to patients with CD (Figures 2C–F). HS-NM had a significantly higher abundance of the *Ruminococcus cluster* and the *Lachnospiraceae cluster* than patients in the HS-CDM group (Figures 4F–G). Likewise, HS-CDM was observed to have a significantly higher level of the disease associated *Pathogen cluster 1* and *Pathogen cluster 2* (Figures 4H–I). These observations confirm that some patients with HS have a microbiota configuration that is strikingly similar to that of patients with CD and it builds upon our previous work identifying differential microbiome trajectories in HS.

### 3.5. Different microbiota configurations in HS can be replicated in a published dataset

To test if we could validate our findings, we obtained the only other publicly available 16S rRNA gene amplicon faecal microbiota dataset (Lam et al., 2021). We analysed faecal microbiota data from 17 individuals with HS obtained from Lam et al. (2021), in a pilot study that was conducted in the Netherlands. We used the same approach as this current study, calculating the relatedness between samples using ranked genera abundances and subsequent Ward2 clustering (Supplementary Figure 8). Using this method, we were able to identify two distinct microbiome clusters in line with our earlier findings (referred to as Lam-HS-NM and Lam-HS-CDM). First, we wanted to compare the abundance of genera which were associated with a normal microbiota composition between the two clusters. We found that the Lam-HS-NM patient group had significantly higher levels of “normal” genera, as identified in Figure 1C (when comparing controls to patients with CD), than the Lam-HS-CDM group (Supplementary Figure 9). Patients in the Lam-HS-CDM group had significantly higher levels of genera associated with CD (also identified in Figure 1C; Supplementary Figure 10). This analysis further validates the existence of two different microbiota configurations in patients with HS.

### 3.6. Medication and diet metadata associate with differences in microbiota composition in HS

**Figure 5:**
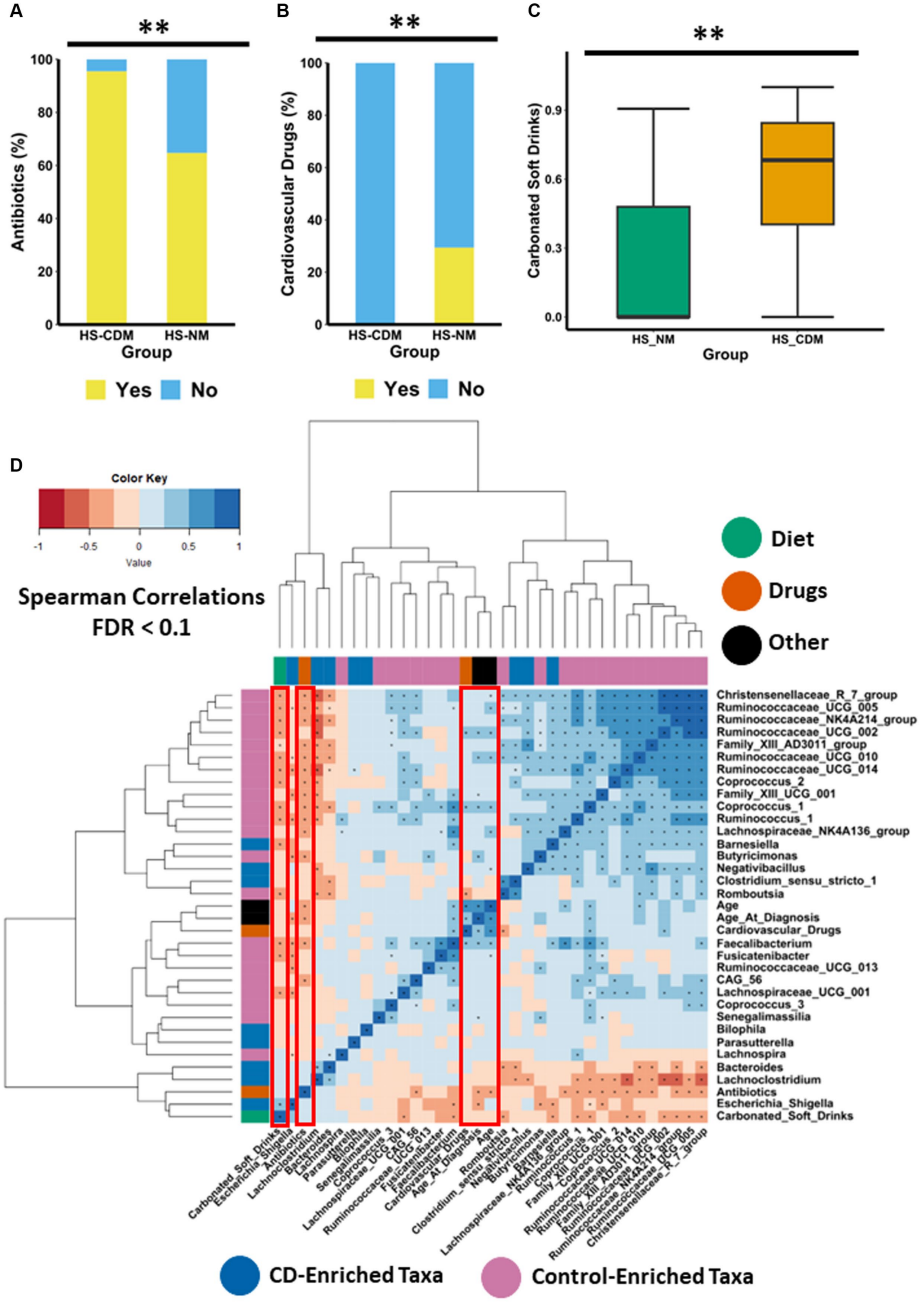
Drugs and diet associate with differences in microbiota composition in HS. Stacked barplot showing the consumption levels of **(A)** antibiotics and **(B)** cardiovascular drugs between the HS-NM and HS-CDM groups. Significance was tested using Fishers exact test. **(C)** Boxplot showing the daily frequency of consumption of carbonated soft drinks between the groups. Significance was tested using a Wilcoxon test. The annotations used for *p*-values are *p* < 0.05*; *p* < 0.01**; *p* < 0.001***. All displayed *p*-values are FDR corrected. **(D)** Heatmap showing correlations between specific taxa and age, age at diagnosis, antibiotics, carbonated soft drinks and antibiotics. The taxa used in this correlation analysis were identified as the top 30 discriminatory features from machine learning random forest classifier comparing controls to CD. Significance was assumed at <0.1* and all *p*-values are FDR corrected.

Given the wide range of factors known to influence microbiota composition, we wanted to establish whether any clinically relevant metadata (including demographics, disease severity, treatment history, drug consumption and habitual diet) differed between the HS-NM and HS-CDM patients groups. The data available for this analysis is presented in Supplementary File 5. We found that patients in the HS-NM group were significantly older than individuals in the HS-CDM group (Supplementary Figure 11A). In addition, we also discovered that the HS-NM group were significantly older at the time of HS diagnosis than patients in the HS-CDM group (Supplementary Figure 11B). Naturally, both factors (age and age of diagnosis) strongly correlated (positive) with one another (*p* = 0.003). Hurley score which is a measure of disease severity in HS was not detected as being significantly different between the groups. In fact, when we compared the microbiota composition between the different levels of severity in HS (3 being the most severe) using PCoA of Bray–Curtis dissimilarity, we did not detect any significant differences (Supplementary Figure 12). Thus, disease severity in HS is not associated with differences in faecal microbiota composition. Antibiotic usage (within the last year) was significantly higher in the HS-CDM patient group compared to HS-NM (Figure 5A). Given that specific species of skin microbiota may drive inflammation in HS, antibiotic treatment is one of the most common first line treatments for management of the disease. Interestingly, a number of patients from both groups were taking antibiotics at the time of sample collection (Supplementary File 5), although no significant difference in current use could be detected between the groups. Although other treatments used to manage HS in this study population are known to influence microbiota composition, including anti-TNFα therapy (infliximab), immunomodulator therapy and surgery, we could not detect any differences in the number of patients between the HS-NM and HS-CDM group using these treatments.

Next, we focused on identifying secondary drugs (i.e., drugs that are not directly used to treat HS such as antibiotics) whose consumption co-varied between the HS-NM and HS-CDM study groups. In order to do so effectively we only wanted to examine informative variables which had a large effect size. Thus, we conducted logistic regression on intake data for medications which were consumed in 20% or more of the study population (removing those with a small effect size), whilst adjusting for a number of demographic (gender, age, age at diagnosis) and clinically important characteristics (disease severity and treatment history; Supplementary File 6). Using this approach, we identified a group of cardiovascular drugs as significantly co-varying with differences in faecal microbiota composition in patients with HS (Table 2). This group of drugs includes aspirin, statins, beta-blockers, angiotensin II receptor antagonists and angiotensin-converting enzyme (ACE) inhibitors, all of which are used to treat cardiovascular disease. Furthermore, when we compared the consumption levels of cardiovascular drugs between the groups, we found it was significantly higher in patients from the HS-NM group (Figure 5B).

**Table 2:** Diet and drug consumption co-varies with differences in microbiota composition.

	Estimate	Std error	Z-value	Pr(>Chi)	Q-value
**Cardiovascular medications**					
Intercept	−1.51972	2.17419	−0.699		
Age	0.04841	0.06289	0.77	0.033877^*^	0.12
Age at diagnosis	−0.06025	0.0666	−0.905	0.041790^*^	0.12
Gender	0.70949	1.01584	0.698	0.774202	0.86
Hurley score	−0.58962	0.65072	−0.906	0.642929	0.82
Surgery for HS	0.97826	0.9454	1.035	0.890866	0.89
Antibiotics (last year)	2.4279	1.24088	1.957	0.049694^*^	0.12
Current anti-TNFα therapy	−1.03846	1.33051	−0.78	0.661523	0.82
Previous anti-TNFα therapy	−0.30019	1.35177	−0.222	0.323765	0.53
Immunomodulator therapy	20.91855	7375.77	0.003	0.09492	0.18
Cardiovascular medications	−20.4486	2805.03	−0.007	0.002749^**^	0.02^*^
**Carbonated soft drinks**					
Intercept	−1.32478	2.0772	−0.638		
Age	−0.00713	0.04909	−0.145	0.029712*	0.09
Age at diagnosis	−0.0633	0.05497	−1.151	0.088597	0.19
Gender	−0.23199	1.19189	−0.195	0.7154	0.89
Hurley score	−0.50905	0.65224	−0.78	0.974009	0.97
Surgery for HS	0.82643	0.99168	0.833	0.836732	0.92
Antibiotics (last year)	2.73648	1.49051	1.836	0.011686*	0.05
Current anti-TNFα therapy	−0.27681	1.17628	−0.235	0.604441	0.86
Previous anti-Tnfα therapy	−2.13611	1.46653	−1.457	0.211059	0.35
Immunomodulator therapy	19.28246	2732.849	0.007	0.097685	0.19
Carbonated soft drinks	3.55746	1.25323	2.839	0.001033^**^	0.01^*^

The annotations used for *p* values are *p* < 0.05^*^; *p* < 0.01^*^^*^; *p* < 0.001^*^^*^^*^.

To examine the consumption of specific dietary ingredients with respect to differences in microbiota composition in HS (HS-NM and HS-CDM), we also used logistic regression adjusting for demographics (gender, age, age at diagnosis) and clinically relevant metadata (disease severity and treatment history). To do this effectively we removed dietary ingredients which had a small effect size (Cohen’s d < 0.5) from our models. Using this method, we identified one dietary ingredient as co-varying with differences in microbiota composition in HS (Table 2). Specifically, we found that carbonated soft drink consumption was significantly higher in patients in the HS-CDM group than those in the HS-NM group (Figure 5C; Supplementary File 6). Interestingly, no significant difference was observed between HS-NM and HS-CDM for overall habitual diet (Supplementary Figure 13).

To identify host-associated factors that might associate with specific microbiota, we conducted spearman correlation analysis between diet, drugs, age and age at diagnosis with the top 30 genera from a random forest classifier comparing healthy and CD. Interestingly, we found that carbonated soft drink intake had a strong positive correlation with *Escherichia_Shigella* (Figure 5D). Carbonated soft drink consumption in HS was also observed to negatively correlate with 13 different genera identified as being enriched in controls, including *Christensenellaceae_R7_group*, *Coprococcus_2*, *Coprococcus_1*, *Faecalibacterium* and several genera in the *Ruminococcaceae* family (Figure 5D). Cardiovascular drugs were shown to positively correlate with the health associated *Coprococcus_2* and *Ruminococcaceae_UCG_002*. Antibiotics had strong negative associations with 16 genera enriched in the control faecal microbiota (Figure 5D). Interestingly, both age and age of diagnosis had strong positive correlations with numerous health associated genera, whilst the latter was also observed to have a strong negative association with *Escherichia_Shigella* (Figure 5D). To identify how these factors associated with CAGs, we determined spearman correlations (Supplementary Figure 14). As expected, carbonated soft drink consumption negatively correlated with the relative abundance of the *Ruminococcus cluster* and *Lachnospiraceae cluster* (Supplementary Figure 14). Antibiotics were also observed to have a negative association with the *Ruminococcus cluster*. Both cardiovascular drugs and age had a strong negative association with *Pathogen cluster 2*.

### 3.7. Faecal microbiota in HS associates with an inflammatory phenotype

**Figure 6:**
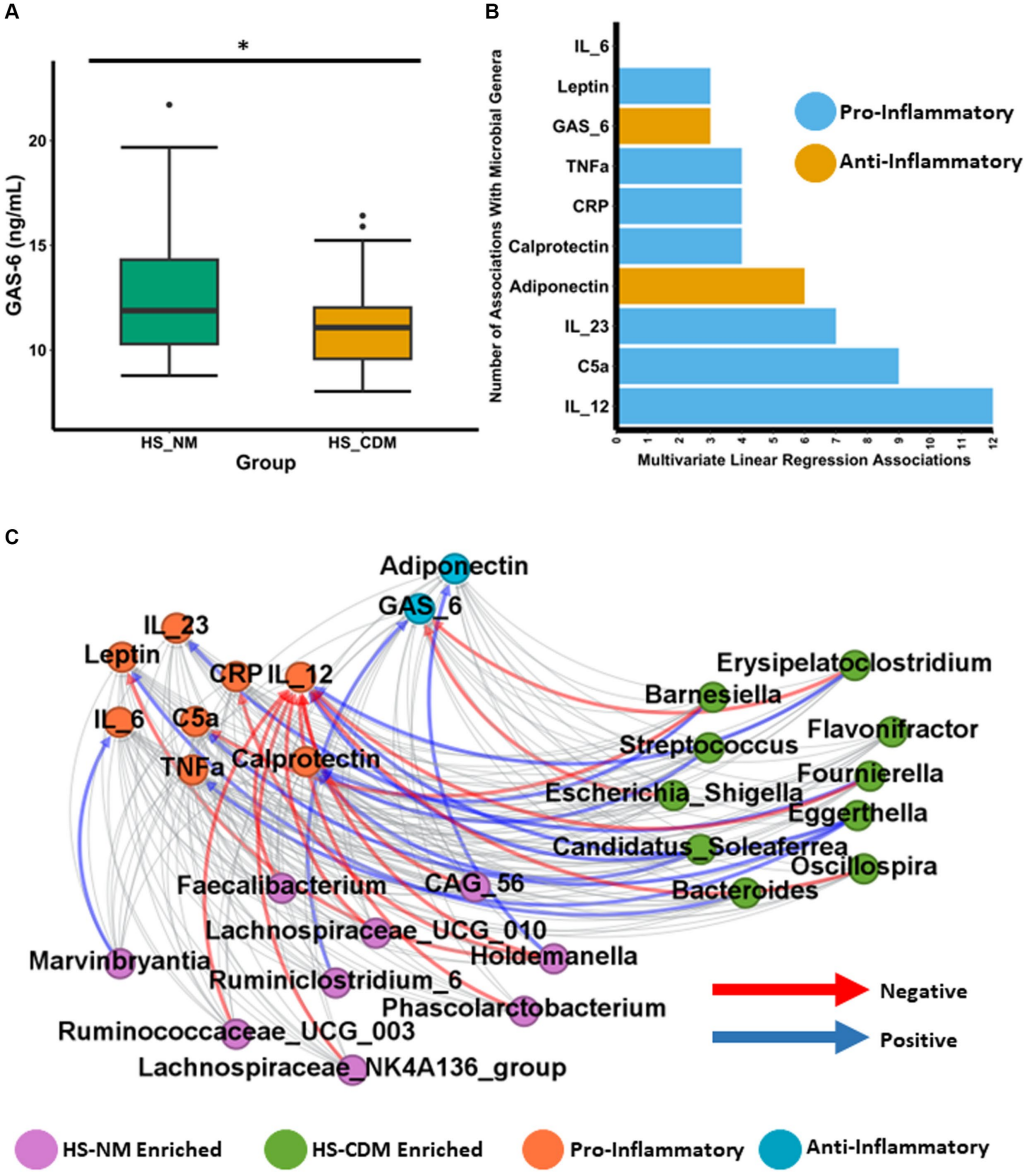
Faecal microbiota in HS associates with an inflammatory phenotype. **(A)** Boxplot showing the levels of Growth Arrest-Specific 6 (Gas6) in serum of patients with HS. Wilcoxon test was used to determine significance. The annotations used for *p*-values are *p* < 0.05 *; *p* < 0.01 **; *p* < 0.001***. **(B)** Barplot showing the number of significant associations (as determined using linear regression) detected between each specific inflammatory marker and general identified as being significantly differentially abundant between the HS-NM and HS-CDM group through ANCOMBC. **(C)** Network plot showing significant correlations between inflammatory markers and specific genera as determined using CCREPE.

Although changes to the microbiota in CD are likely secondary to disease development, there is clear evidence that a number of species enriched in the CD microbiota directly contribute a pro-inflammatory effect maintaining intestinal inflammation and disease progression (Sartor, 2008; Baker et al., 2014; Schirmer et al., 2019; Candelli et al., 2021; Pavel et al., 2021). Given that we detected a minority of patients with HS as having a microbiota composition similar to CD, we hypothesised that inflammatory phenotypes might also differ between the HS-NM and HS-CDM patients groups. To test this hypothesis, we quantified the concentration of 10 different inflammatory markers from serum in patients with HS. This included several cytokines (IL-12, IL-23, IL-6 and TNFα) as well as the adipokines leptin and adiponectin (Supplementary File 7). We also measured C-reactive protein (CRP), complement component 5a (C5a) and the anti-inflammatory, the vitamin-k dependent protein, Growth Arrest-Specific gene 6 (Gas6; Supplementary File 7). Furthermore, we also quantified the intestinal inflammatory marker faecal calprotectin which is commonly used as a detection method for CD (Vernia et al., 2020). When we compared the levels between patients in HS-NM and HS-CDM for all 10 markers of inflammation only Gas6 was found to be significantly different (Figure 6A and data not shown). Serum Gas6 (ng/mL) levels were significantly higher in patients in with a healthy-like microbiota composition (HS-NM; Figure 6A).

Next, we wanted to establish whether the levels of inflammation associated with the abundance of specific gut microbiota genera in patients with HS. To do so we conducted linear regression between the 10 inflammatory markers and the 60 genera which were identified as being significantly differentially abundant between the HS-NM and HS-CDM groups in Figure 4E. The complete results are shown in Supplementary File 7. Our models revealed a total of 55 different associations between genera abundance and inflammation in HS (Figure 6B). IL-12 which is an important cytokine in CD development had the most associations (12) of any inflammatory marker, followed by C5a (9) and IL-23 (7) (Figure 6B). In order to further investigate this relationship, we conducted CCREPE (Compositionality Corrected by REnormalization and PErmutation) analysis between the ranked inflammatory markers and genera abundances. We identified a number of associations between inflammation and taxa associated with CD (Figure 6C). IL-12 serum levels were found to positively correlate with the HS-CDM enriched *Eggerthella*, *Candidatus_Solefera* and *Erysipelatoclostridium* (Figure 6C; Supplementary File 8). IL-12 also negatively correlated with the abundance of 9 health associated genera enriched in the HS-NM patient group. This included *Faecalibacterium*, *Holdemanella*, *Lachnospiraceae_NK4A136_group*, *Lachnospiraceae_UCG_010* and *Ruminococcaceae_UCG_003*. C5a was also found to have a positive association with *Eggerthella* whilst calprotectin levels positively correlated with *Streptococcus* and *Candidatus_Solefera*. Adiponectin which is thought have be anti-inflammatory showed a positive association with the HS-NM enriched *Holdemanella* (Figure 6C; Supplementary File 8). The opposite was observed for both CRP and Leptin levels which had strong negative correlations with this health-associated genera. Interestingly, leptin also had a strong positive association with the HS-CDM enriched Bacteroides genera. Levels of Gas6 were found to co-vary with differences in microbiota composition in HS and they positively correlated with the abundance of the health associated *Ruminiclostridium_6*. Gas6 was also detected as negatively correlating with two CD associated genera, *Escherichia_Shigella* and *Erysipelatoclostridium*. In summary, the differences in faecal microbiota compositions detected were associated with different levels of inflammatory markers in patients with HS. These findings highlight that the HS-CDM microbiota associates with a greater inflammatory phenotype.

## 4. Discussion

In this current study we report that some patients with HS have a faecal microbiota configuration characteristic of CD. However, the majority of patients with HS were detected as having a “normal” microbiota composition most similar to controls. Antibiotics, which are a common first line treatment for HS, were a key covariate of distinct microbiota compositions in HS. We also detected several associations between the microbiota and inflammation levels in patients with HS, including IL-12, which is directly implicated in CD pathogenesis. These findings highlight the potential of the faecal microbiota as a biomarker in identifying patients with HS at risk for development of CD.

Specifically, we reported that the microbiota in 40% of patients with HS was enriched with genera such as *Escherichia_Shigella, Veillonella* and *Enterococcus* which is characteristic of CD. Several species belonging to these genera are known to promote intestinal inflammation. For example, adherent invasive *Escherichia coli* can produce outer membrane vesicles which increases the secretion of pro-inflammatory cytokines (Barnich and Darfeuille-Michaud, 2007; Eaves-Pyles et al., 2008; Rolhion et al., 2010; Kim et al., 2013; Agus et al., 2014; Kaparakis-Liaskos and Ferrero, 2015; Lee et al., 2019; Mirsepasi-Lauridsen et al., 2019). *Enterococcus faecalis* can secrete metalloproteases (Steck et al., 2011; Zhou et al., 2016) which degrade the intestinal mucosa, an important event in CD pathogenesis (Marônek et al., 2021). Higher abundance of the oral bacteria *Veillonella parvula* which is common in CD, is directly linked with higher levels of the pro-inflammatory compound nitrate which promotes colonisation by this species (Rojas-Tapias et al., 2022). We also reported a depletion of beneficial microbiota (*Faecalibacterium* spp.) in patients with HS who had a CD microbiota configuration. Some of these taxa may exert a protective effect for the host. For example, *Faecalibacterium prausnitzii* secretes an anti-inflammatory protein which can inhibit the NF-κB pathway in intestinal epithelial cells (Quévrain et al., 2016). Our findings suggest that patients with HS who have a CD microbiota configuration may be at risk for higher levels of microbiota induced intestinal inflammation than patients with a microbiota composition resembling controls.

Drug consumption and diet were identified as important covariates of compositional differences in patients with HS. We identified that antibiotic use negatively associated with the abundance of several health associated microbiota in patients with HS. Antibiotics are directly associated with the risk of new-onset CD development (Ungaro et al., 2014; Theochari et al., 2018). Specifically, antibiotic use has been associated with lower numbers of protective commensals which can open a niche for typically low abundant pathogenic species (Modi et al., 2014; Haak et al., 2019). Cardiovascular drug consumption was higher in patients with HS who have a “normal” microbiota composition. The group of drugs have been shown to significantly alter microbiota composition, including ACE inhibitors (Vich Vila et al., 2020), beta-blockers (Weersma et al., 2020), angiotensin II receptor antagonists (Zhernakova et al., 2016), aspirin (Prizment et al., 2020) and statins (Vieira-Silva et al., 2020). Interestingly, the *Bacteroides2* enterotype which is characterised by a high abundance of *Bacteroides* and low abundance of *Faecalibacterium* has been associated with systemic inflammation and is found in high levels in patients with IBD (Vandeputte et al., 2017; Vieira-Silva et al., 2019). An important study examining this inflammatory enterotype (*Bacteroides2*) in obesity showed that statin treatment negatively correlated with its abundance suggesting a beneficial role for this drug in the context of the microbiome (Vieira-Silva et al., 2020). Although no differences were reported in overall habitual diet between HS microbiome groups, we detected a significantly higher consumption of carbonated soft drinks in individuals with a CD-like microbiota configuration. High intake of carbonated soft drinks was reported to increase risk of CD development (Yang et al., 2019). Key ingredients of carbonated soft drinks include simple sugars (glucose, sucrose and fructose) and emulsifiers (Vilela et al., 2018). Both of these ingredients have independently been shown to alter microbiota composition and exacerbate inflammation in murine colitis models (Chassaing et al., 2015; Khan et al., 2020).

We also report significantly lower levels of the Gas6 protein in patients with HS who have a CD microbiota configuration. Gas6 interacts with TAM receptors maintaining immune homeostasis through TLR signalling (Lemke, 2013; Rizzi et al., 2023). Gas6 can exert an anti-inflammatory through activation of TAM receptors present on activated T regulatory cells (Lemke and Rothlin, 2008; Sainaghi et al., 2013; Bellan et al., 2016; Cohen et al., 2019; Rizzi et al., 2023). Interestingly, Gas6^−/−^ mice exhibited more severe DSS-induced colitis (Akitake-Kawano et al., 2013). We also observe negative correlations between the levels of several putatively beneficial microbiota and the pro-inflammatory cytokine IL-12 in patients with HS. This is important as IL-12 promotes inflammation in CD by regulating the differentiation of naive CD4+ T cells into IFNγ-producing TH1 cells (Macdonald et al., 2016; Moschen et al., 2018; Petagna et al., 2020). Our findings indicate that patients with HS who have low levels of health associated taxa are more likely to have higher levels of IL-12 mediated inflammation.

Diet in HS was characterised by high level consumption of ingredients high in sugar and saturated fat, which is a staple of the Western diet. Consumption of food items high in dietary fibre (such as fruit and vegetables) were least associated with the typical dietary pattern in patients with HS. Interestingly, habitual diet in HS was strikingly similar to patients with CD. HS and CD subjects differed in their consumption of a small number of dietary ingredients, but these food items included sugar (differently consumed in CD) and carbonated soft drinks (differentially consumed in HS) the macronutrient composition of which is similar. Ultimately these findings highlight the need for greater nutritional support for patients with HS. Structured dietary advice and/or dietary intervention represents a promising avenue to alleviate disease burden. Diets high in fibre have anti-inflammatory potential which may help improve symptoms of HS.

One limitation of this study was that previously published data available to validate the findings was limited. Lam et al. (2021) analysed only a small number of samples which we could avail of to conduct replication analysis. A large-scale comparative study will be required to consolidate the findings of this study. Furthermore, comparing the skin microbiota between patients with HS with either a normal or CD-like microbiota configuration was not possible due to the small number of samples available at different dermatological sites. A future study will be conducted to determine whether the findings from this study also extend to the skin microbiome.

Whether alterations in the gut microbiota are a consequence or cause of intestinal inflammation remains to be determined. However, it is clear that intestinal inflammation and changes in oxygen tension can cause major disturbances in microbiota composition. Theoretically, patients with HS identified as having a CD-type microbiota configuration might be at greater risk for development of CD. Prospective longitudinal sampling of the faecal microbiota in HS might enhance our understanding of the role of the intestinal microbiota in the pathogenesis of HS and clarify direct or indirect links with risk of developing CD.

## Data availability statement

Publicly available datasets were analysed in this study. This data can be found at: European Nucleotide Archive (ENA) under accession number PRJEB43835 and PRJNA414072.

## Ethics statement

Ethical approval was not required for the studies involving humans because the data used was obtained from previously published studies. The studies were conducted in accordance with the local legislation and institutional requirements. The human samples used in this study were acquired from primarily isolated as part of your previous study for which ethical approval was obtained. Written informed consent to participate in this study was not required from the participants or the participants’ legal guardians/next of kin in accordance with the national legislation and the institutional requirements.

## Author contributions

PC: Data curation, Formal analysis, Validation, Visualization, Writing – original draft, Writing – review & editing. SM: Data curation, Writing – review & editing. CH: Formal analysis, Writing – review & editing. TG: Conceptualization, Writing – review & editing. JC: Conceptualization, Writing – review & editing. A-MT: Data curation, Writing – review & editing. MM: Data curation, Writing – review & editing. EO’C: Conceptualization, Writing – review & editing. FS: Writing – review & editing. PO’T: Conceptualization, Supervision, Writing – review & editing.
