# Supplementary material for: Comparative diet-gut microbiome analysis in Crohn’s disease and Hidradenitis suppurativa
Source: Front Microbiol. 2023 Nov 10;14:1289374. doi: 10.3389/fmicb.2023.1289374 (PMC10667482; doi:10.3389/fmicb.2023.1289374)
Supplement: Supplementary file 1 [file Data_Sheet_1.docx]

**
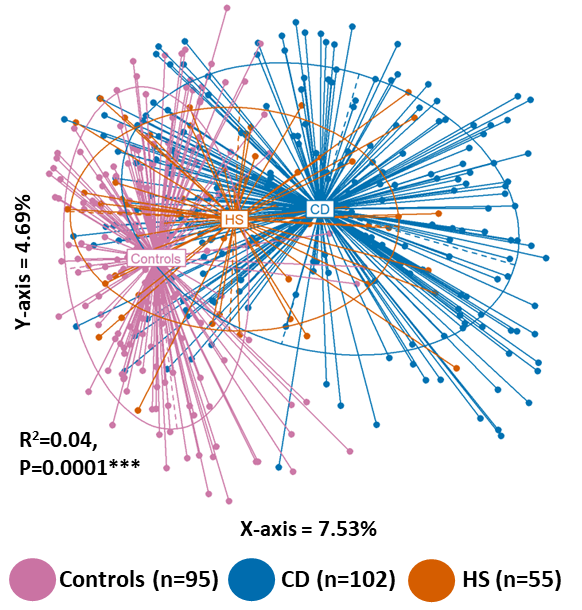
**

**Supplementary Figure 1:** Microbiota composition in HS is significantly different to CD for most individuals. (A) Principal Component Analysis (PCoA) of β-diversity (Bray-Curtis dissimilarity) at the ASV level (16S rRNA gene amplicon profiles). The P Value (0.0001) obtained using a PERMONOVA shows there is statistically significant microbiome separation between the groups even after controlling for the study effect and patient identifier as confounders. The eigen values are also reported which show the variation reported in the X-axis (7.53%) and Y-axis (4.69%) of the PCoA.

**
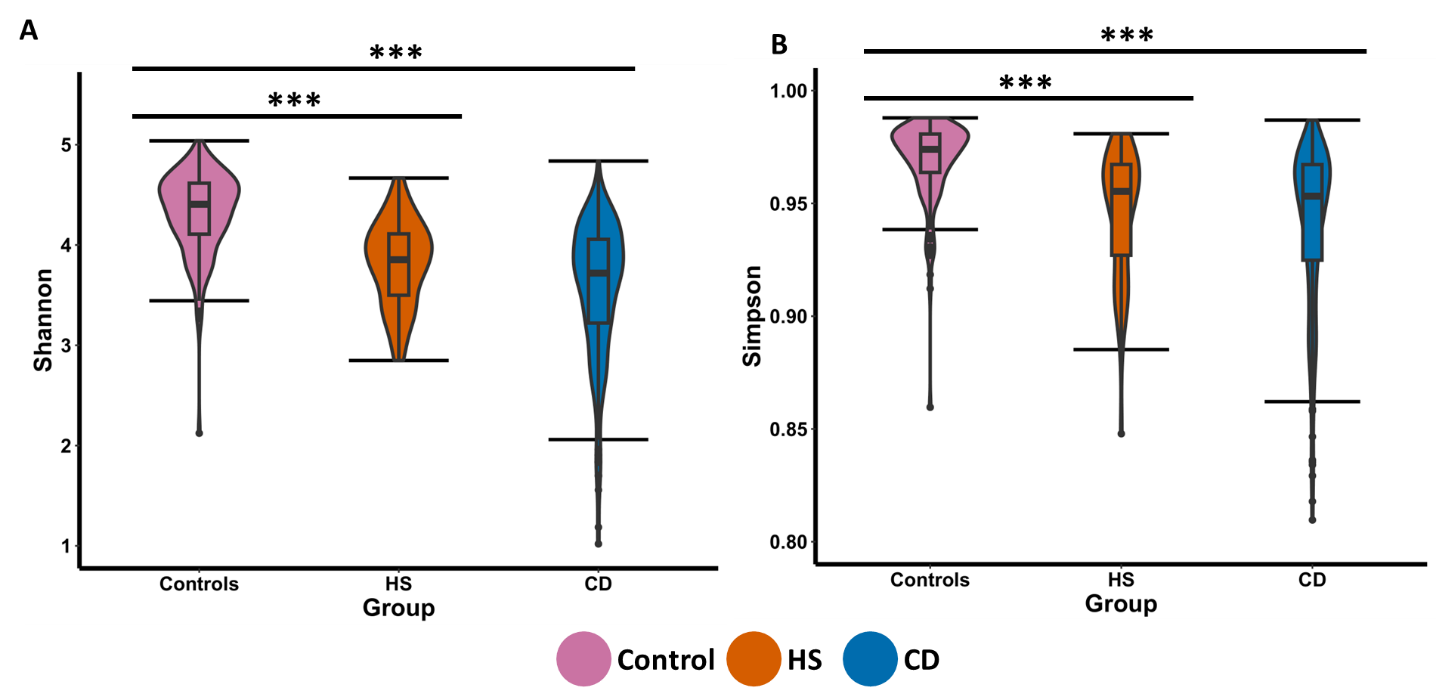
**

**Supplementary Figure 2:** No significant difference in microbiota α-diversity between HS and CD for (A) Shannon and (B) Simpson. The annotations used for P values are P < 0.05 *; P < 0.01 **; P < 0.001***. All displayed P values are FDR corrected.


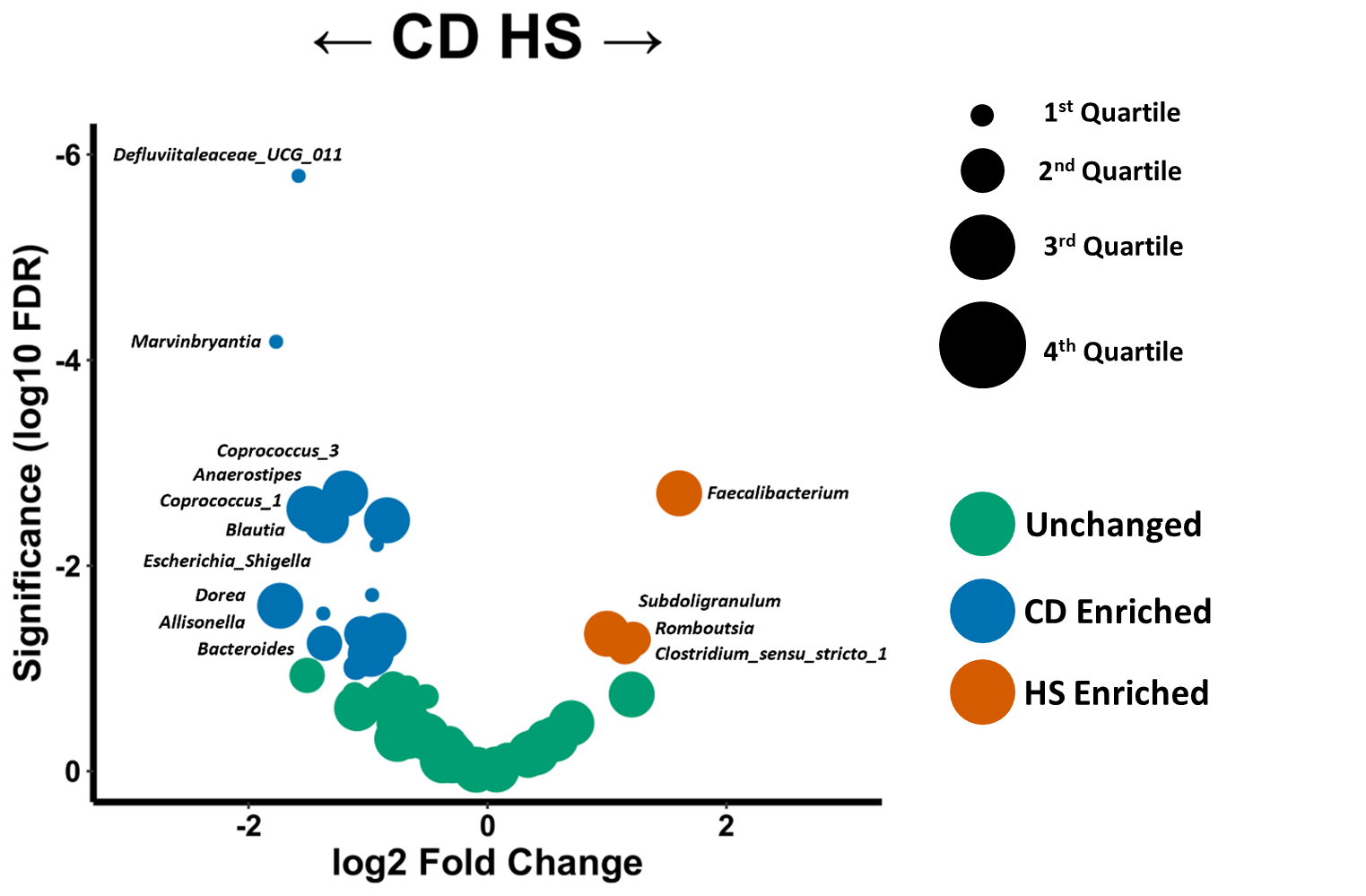


**Supplementary Figure 3:** Microbiota Composition in HS is Significantly Different to CD in some individuals. Volcano Plot showing the most significantly differentially abundant genera between HS and CD as identified through ANCOMBC. The X-axis show the log2 fold change while the Y-axis shows the significant level (log10 FDR). The size of each point refers to the abundance of the species across the group.

**
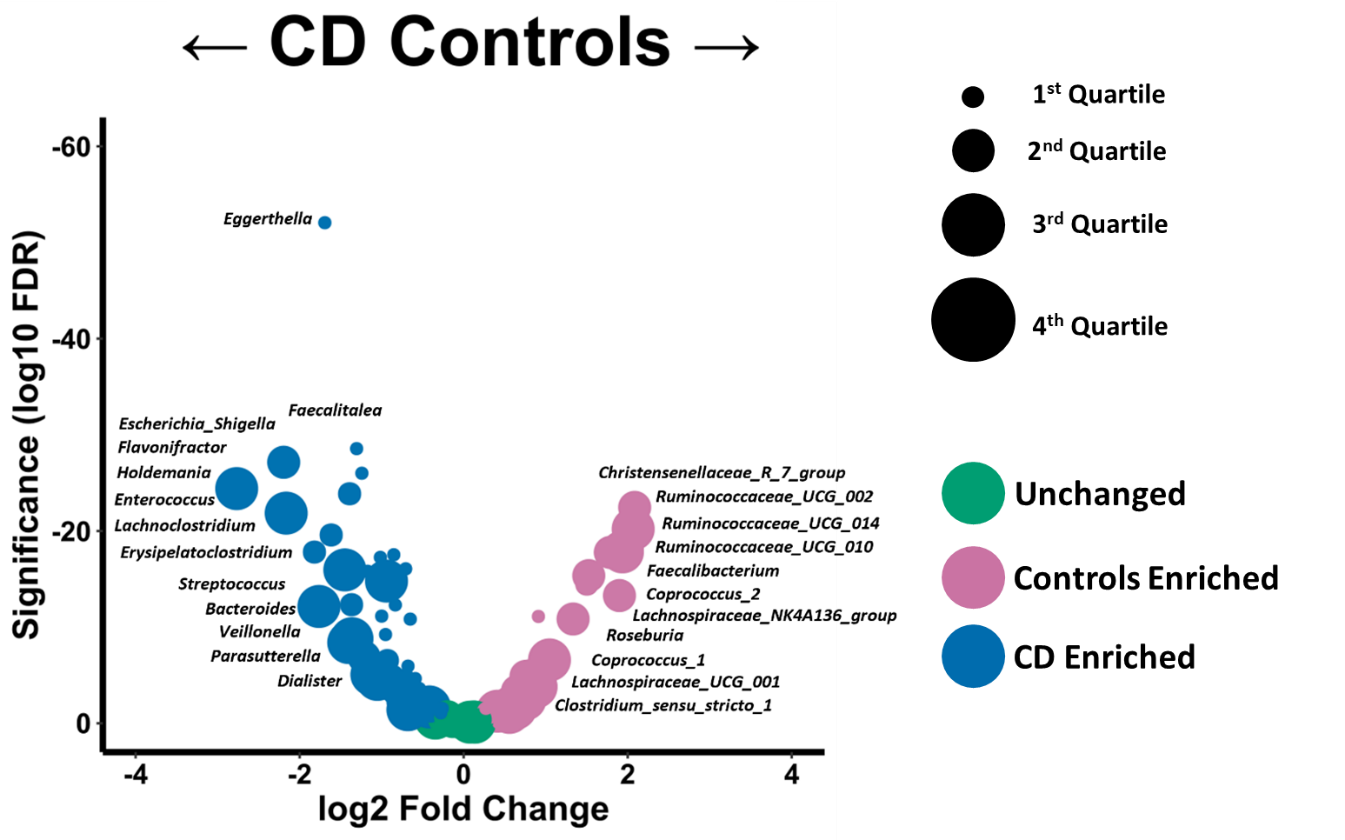
**

**Supplementary Figure 4:** Microbiota Composition in CD is Significantly Different to Controls. Volcano Plot showing the most significantly differentially abundant genera between CD and controls as identified through ANCOMBC. The X-axis show the log2 fold change while the Y-axis shows the significant level (log10 FDR). The size of each point refers to the abundance of the species across the group.

**
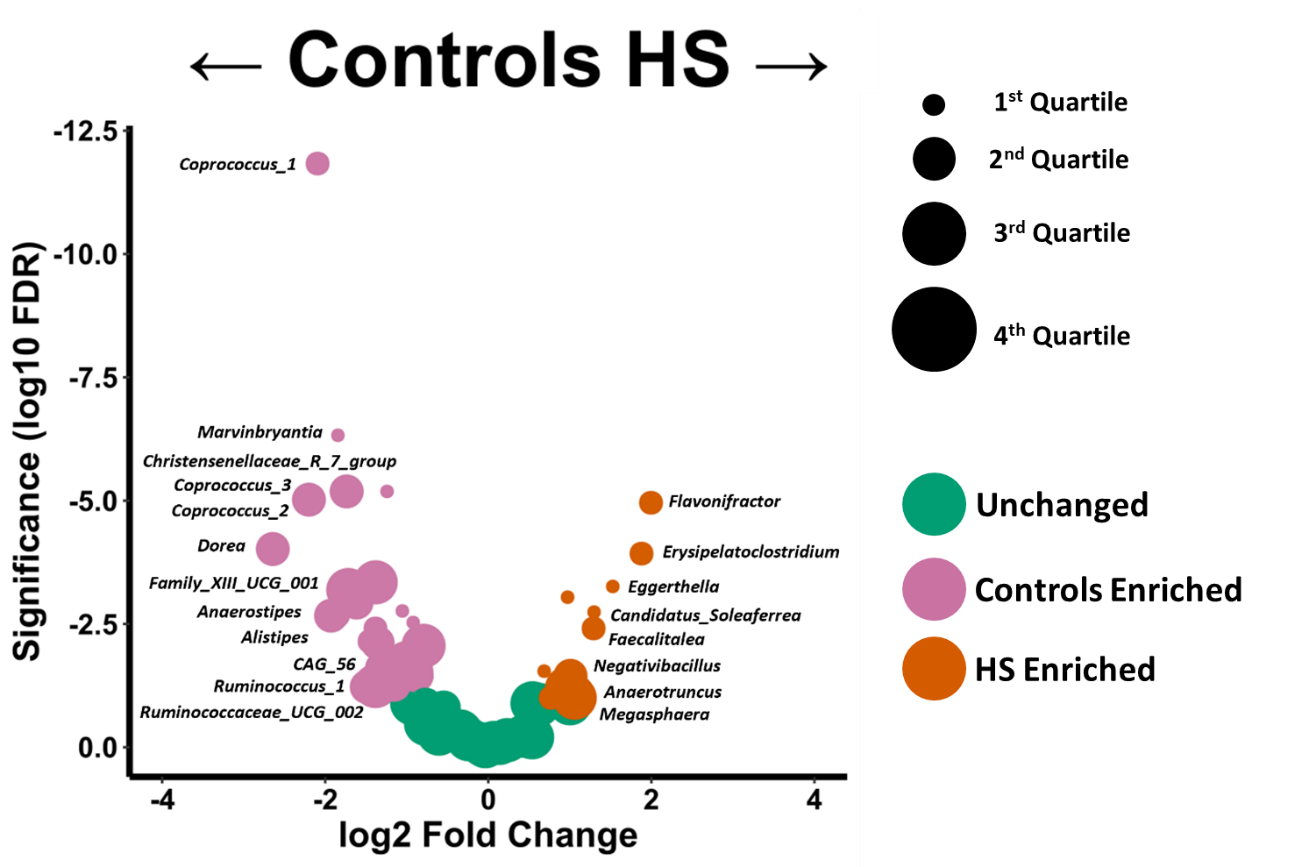
**

**Supplementary Figure 5:** Microbiota Composition in HS is Significantly Different to Controls. Volcano Plot showing the most significantly differentially abundant genera between HS and controls as identified through ANCOMBC. The X-axis show the log2 fold change while the Y-axis shows the significant level (log10 FDR). The size of each point refers to the abundance of the species across the group.

**
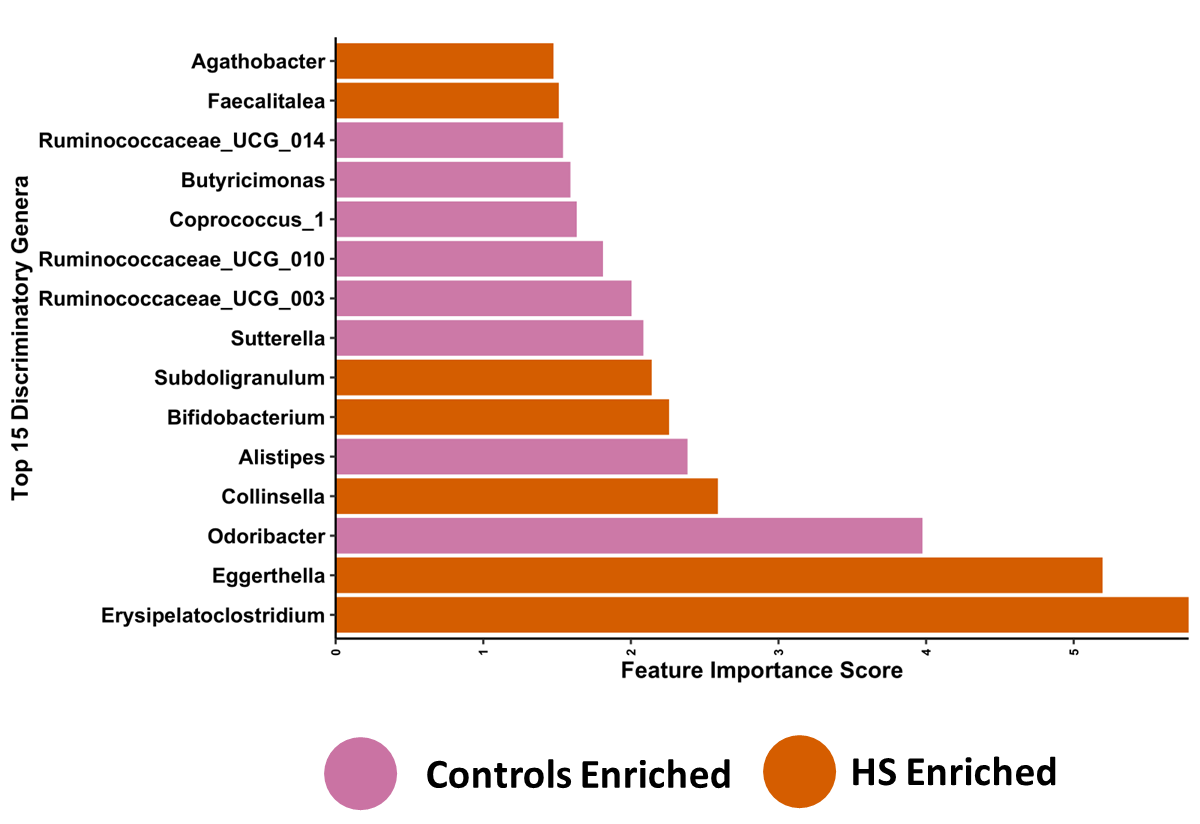
**

**Supplementary Figure 6:** Barplot depicting the top 15 most discriminatory genera from a machine learning random forest classifier comparing HS and controls. The color indicates whether that genera is found in higher abundance in HS or controls. Adjacent table shows the confusion matrix obtained from the random forest classifier. The error rate of the overall model as well as error rate and number of fecal microbiota samples classified into each group is shown.

**
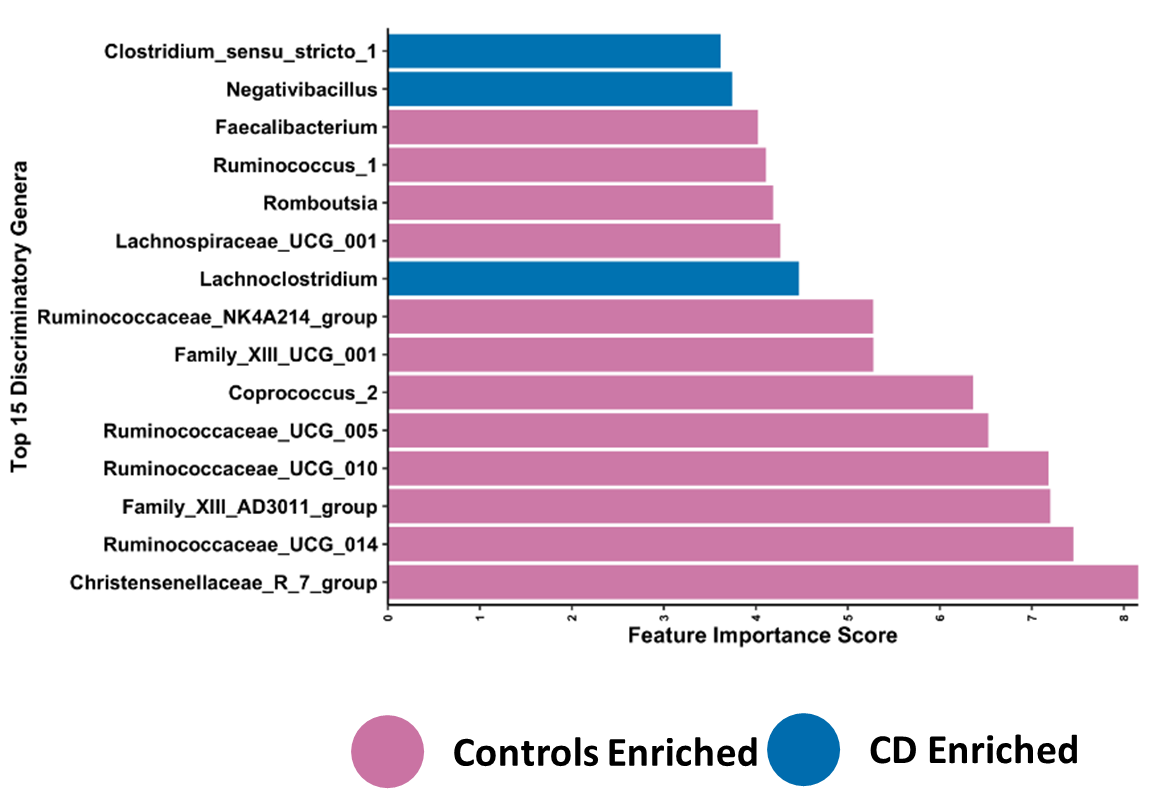
**

**Supplementary Figure 7:** Barplot depicting the top 15 most discriminatory genera from a machine learning random forest classifier comparing CD and controls. The color indicates whether that genera is found in higher abundance in CD or controls. Adjacent table shows the confusion matrix obtained from the random forest classifier. The error rate of the overall model as well as error rate and number of fecal microbiota samples classified into each group is shown.


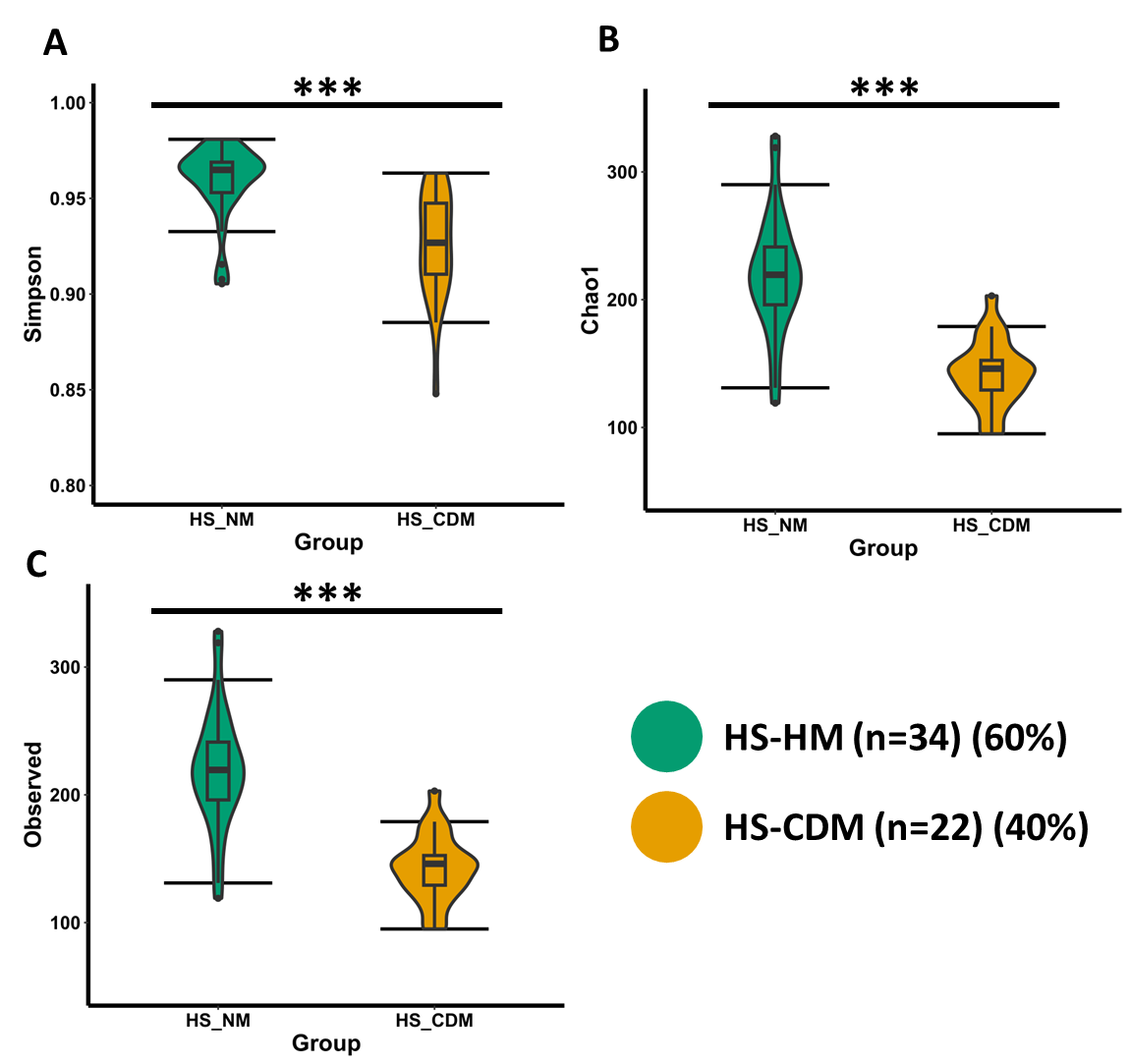


**Supplementary Figure 8:** Microbiota α-diversity is significantly different between HS-NM and and HS-CDM for (A) Simpson and (B) Chao1 and (C) Observed Species. The annotations used for P values are P < 0.05 *; P < 0.01 **; P < 0.001***. All displayed P values are FDR corrected.

**
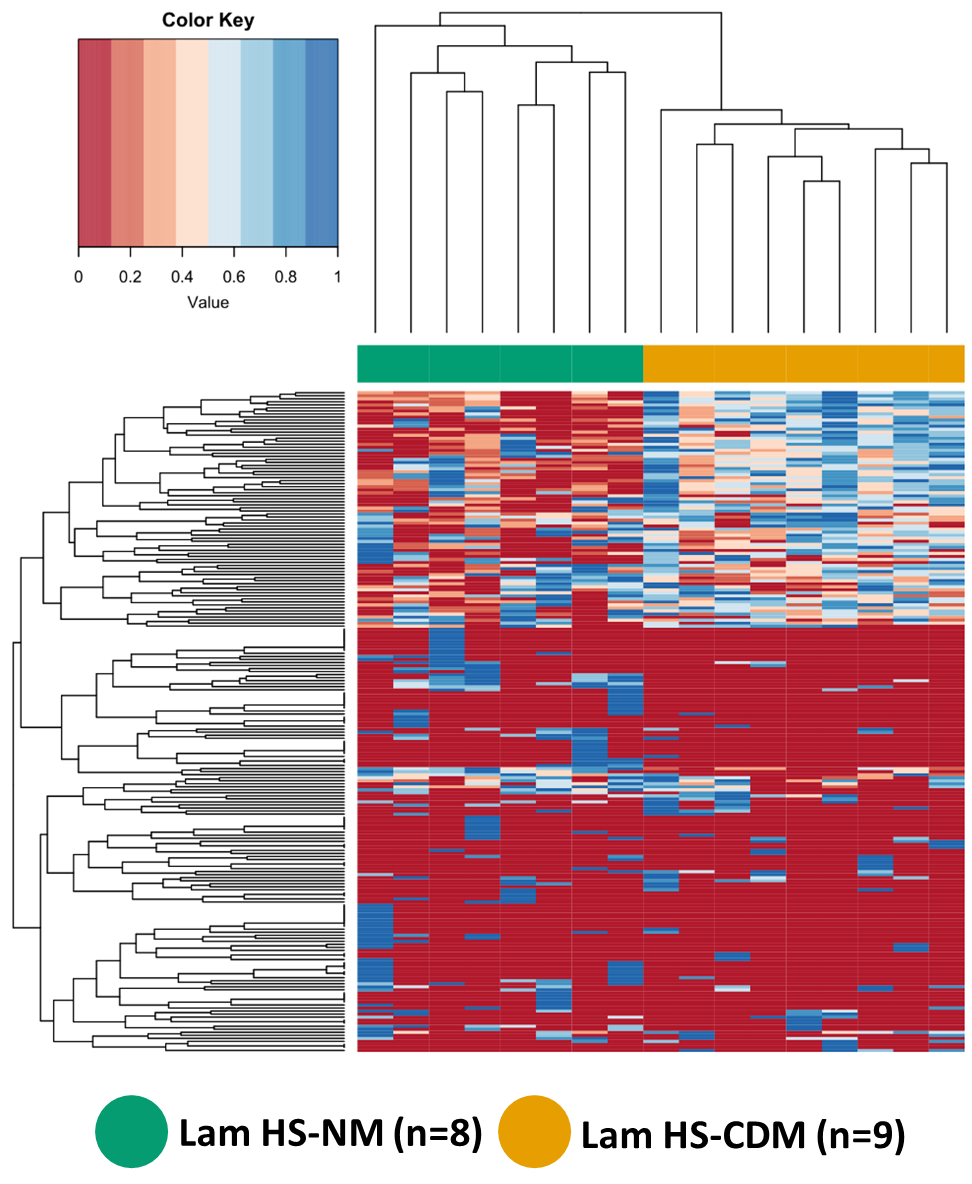
**

**Supplementary Figure 9:** Differential Microbiota Compositions in HS Can Be Replicated in Another Previously Published Dataset. Heatmap showing the genus profiles of all patients with HS from Lam *et al* (2020). These groups were identified through ward.d2 clustering of rank normalised genera abundances and named Lam-HS-NM (green) and Lam-HS-CDM (yellow) as annotated by legend below the heatmap.


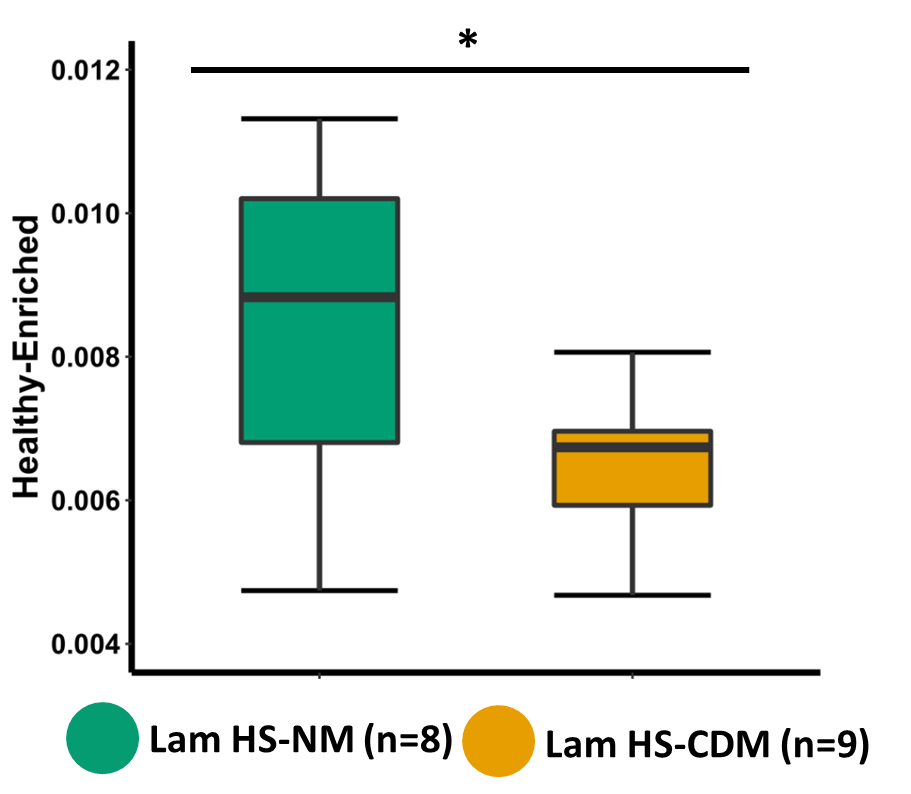


**Supplementary Figure 10:** Differential Microbiota Compositions in HS Can Be Replicated in Another Previously Published Dataset. Boxplot showing the combined abundance of genera identified as being enriched in the control group (when compared to CD as determined in Figure 1C) in patients clusters identified in the Lam *et al* (2020) dataset.


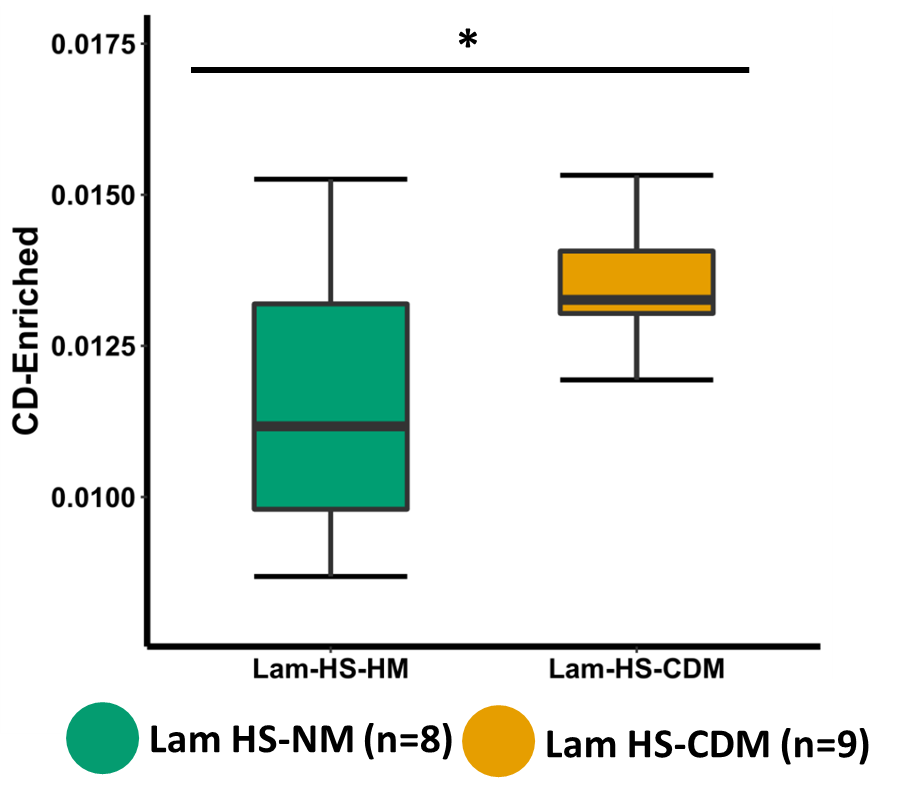


**Supplementary Figure 11:** Differential Microbiota Compositions in HS Can Be Replicated in Another Previously Published Dataset. Boxplot showing the combined abundance of genera identified as being enriched in the CD group (when compared to controls as determined in Figure 1C) in patients clusters identified in the Lam *et al* (2020) dataset.


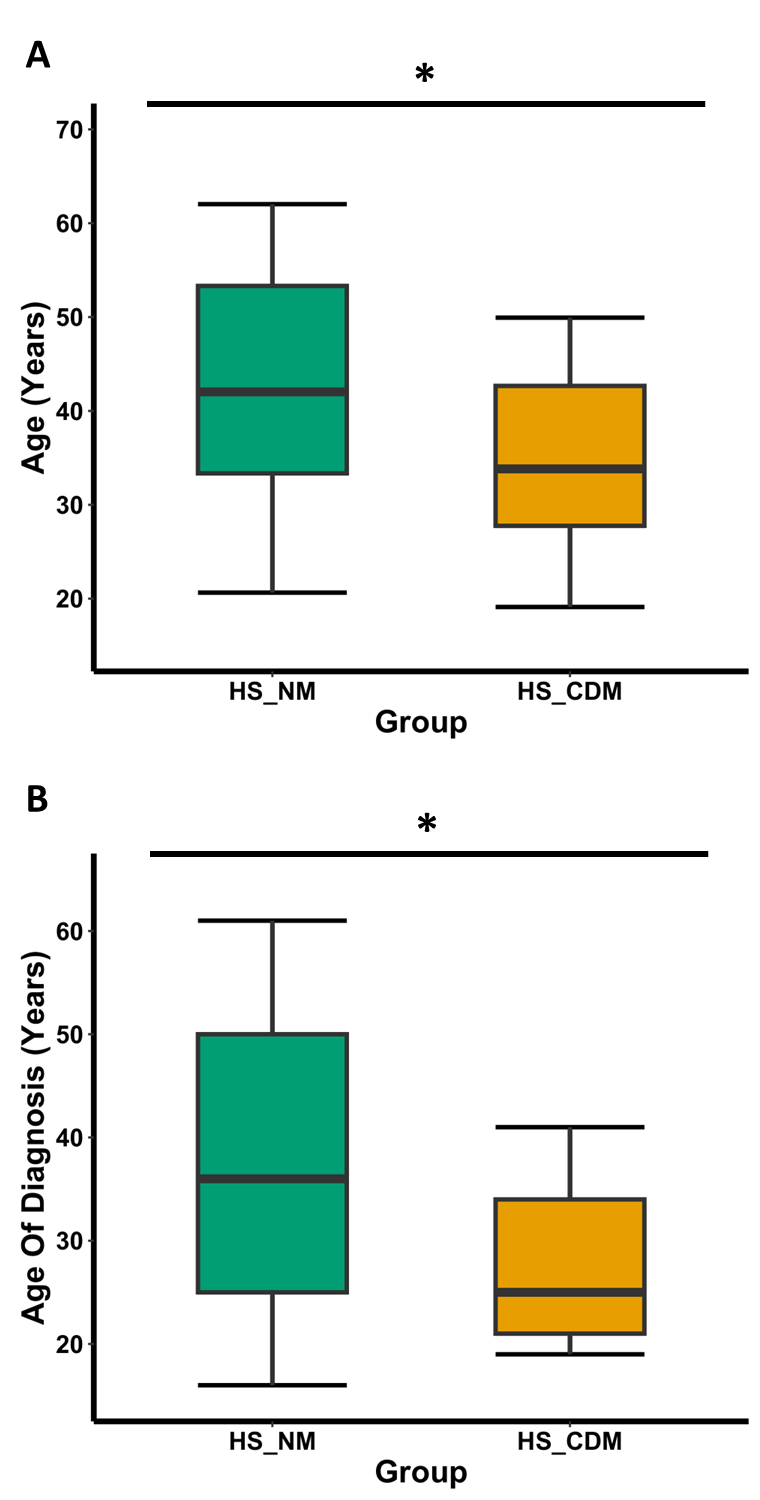


**Supplementary Figure 12:** Patients in the HS-NM patients group are significantly older in age than those in the HS-CDM group. Boxplot showing A) Age and (B) Age of diagnosis. Wilcoxon test was used to determine significance. The annotations used for P values are P < 0.05 *; P < 0.01 **; P < 0.001***. All displayed P values are FDR corrected.


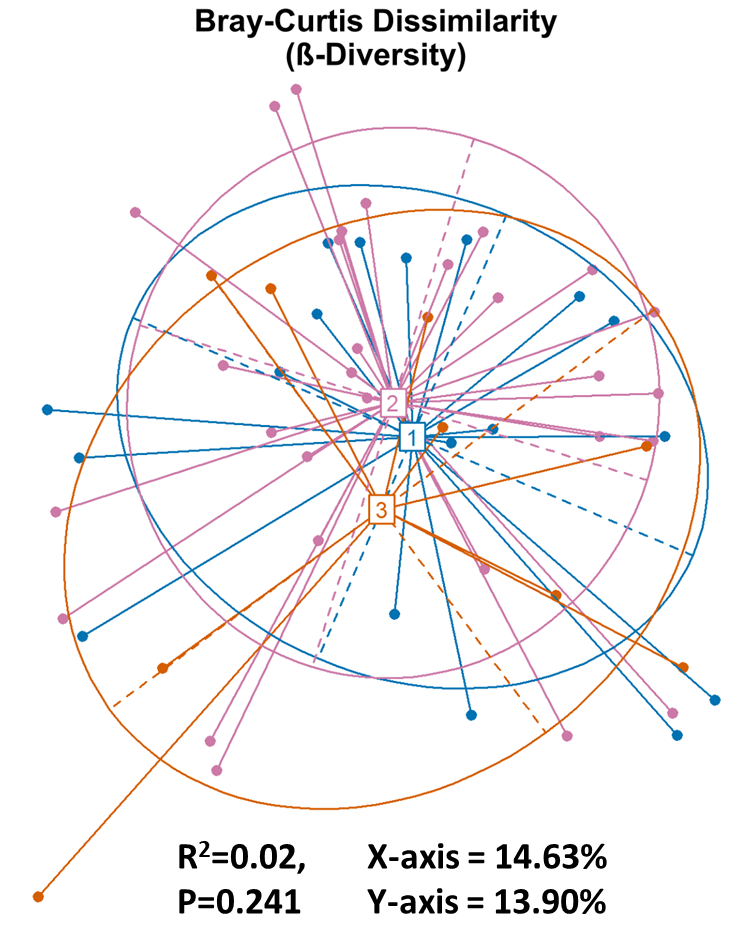


**Supplementary Figure 13:** Disease severity in patients with HS is independent of changes to fecal microbiota composition. Principal Coordinate Analysis (PCoA) of β-diversity (Bray-Curtis dissimilarity) at the genus level (16S rRNA gene amplicon profiles). The P Value (0.241) obtained using a PERMONOVA shows there is no statistically significant microbiome separation between the groups. The eigen values are also reported which show the variation reported in the X-axis (14.63%) and Y-axis (13.90%) of the PCoA.


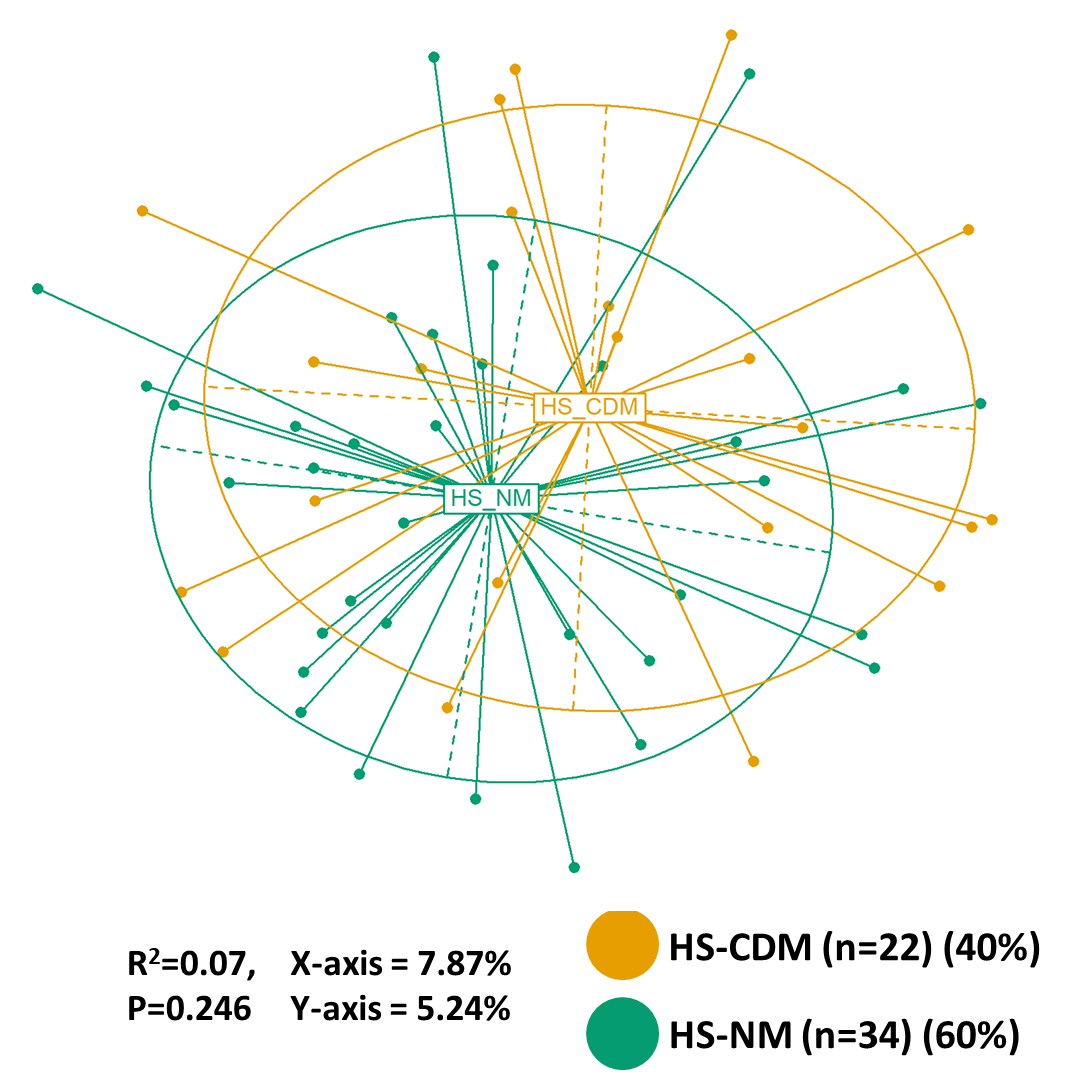


**Supplementary Figure 14:** Overall habitual diet is not significantly different between the HS-NM and HS-CDM patients groups. Principal Coordinate Analysis (PCoA) of Kendall tau distance (dietary profiles). The P Value (0.246) obtained using a PERMONOVA shows there is no statistically significant dietary separation between the groups. The eigen values are also reported which show the variation reported in the X-axis (7.87%) and Y-axis (5.24%) of the PCoA.


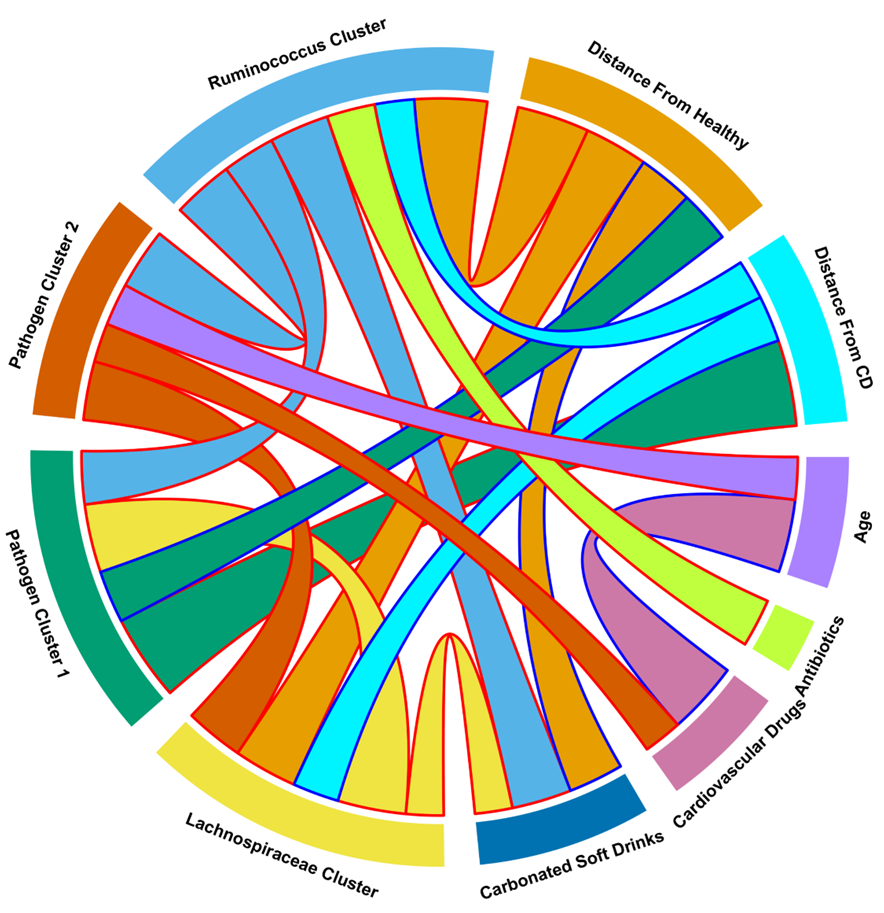


**Supplementary Figure 15:** Chord Diagram representing spearman correlations between CAG abundance and clinically important metadata. Only statistically significant associations are shown (FDR < 0.1). The thickness of each chord is reflective of the correlation coefficient. The outline of each chord highlights whether the association is negative (red) or positive (blue).
